# On the road to universal coverage of postnatal care: considerations for a targeted postnatal care approach for at-risk mother–baby dyads in low-income and middle-income countries informed by a consultation with global experts

**DOI:** 10.1136/bmjopen-2021-058408

**Published:** 2022-06-14

**Authors:** Angela Muriuki, Melanie Yahner, Michael Kiragu, Joseph de Graft-Johnson, Preston Izulla

**Affiliations:** 1 Save the Children, Nairobi, Kenya; 2 Department of Global Health, Save the Children Federation Inc, Washington, DC, USA; 3 Adroitz Consultants Limited, Nairobi, Kenya

**Keywords:** health policy, quality in health care, public health, maternal medicine, community child health

## Abstract

**Introduction:**

The potential of timely, quality postnatal care (PNC) to reduce maternal and newborn mortality and to advance progress toward universal health coverage (UHC) is well-documented. Yet, in many low-income and middle-income countries, coverage of PNC remains low. Risk-stratified approaches can maximise limited resources by targeting mother–baby dyads meeting the evidence-based risk criteria which predict poor postnatal outcomes.

**Objectives:**

To review evidence-based risk criteria for identification of at-risk mother–baby dyads, drawn from a literature review, and to identify key considerations for their use in a risk-stratified PNC approach.

**Design/setting/participants:**

A virtual, semi-structured group discussion was conducted with maternal and newborn health experts on Zoom. Participants were identified through purposive sampling based on content and context expertise.

**Results:**

Seventeen experts, (5 men and 12 women), drawn from policymakers, implementing agencies and academia participated and surfaced several key themes. The identified risk factors are well-known, necessitating accelerated efforts to address underlying drivers of risk. Risk-stratified PNC approaches complement broader UHC efforts by providing an equity lens to identify the most vulnerable mother–baby dyads. However, these should be layered on efforts to strengthen PNC service provision for all mothers and newborns. Risk factors should comprise context-relevant, operationalisable, clinical and non-clinical factors. Even with rising coverage of facility delivery, targeted postnatal home visits still complement facility-based PNC.

**Conclusion:**

Risk-stratified PNC efforts must be considered within broader health systems strengthening efforts. Implementation research at the country level is needed to understand feasibility and practicality of clinical and non-clinical risk factors and identify unintended consequences.

**Strengths and limitations of this study**

- A major strength of this study is the depth and breadth of expertise of the participants in postnatal care (PNC), each bringing a combination of clinical, research, policy and implementation skills across multiple low-income and middle-income countries.
- The consultation brought together experts, many of whom had engaged in parallel discussions around the topic, with the aim of advancing consensus on the role of a targeted PNC approach, and the key considerations of such an approach.
- However, the consultation included a limited number of global experts and did not include mothers, service providers or experts representing Ministries of Health or other government stakeholders as ultimate custodians of a targeted PNC approach.
- In addition, nearly all experts came from a clinical background, which shaped perspectives shared.
- The discussion platform did not allow for confidentiality, which could have led to social desirability bias.

***Angela Muriuki and colleagues*** *argue that there is a critical role for targeted postnatal care (PNC) approaches that prioritise mother–baby dyads who are at risk of poorer outcomes in the postnatal period, given the current low coverage of PNC. However, these approaches must be nested within existing strategies to strengthen provision of PNC for all mothers and babies rather than as stand-alone interventions*.

## Introduction

Approximately 66% of maternal deaths and 75% of neonatal deaths occur within the first week after delivery.^1^ WHO recommends postnatal care (PNC) at a facility, within 24 hours after birth, regardless of place of birth, observation within a facility for at least 24 hours after delivery and early postnatal home visits (PNHVs) by community health workers (CHWs) to complement facility-based PNC.^1^ Despite an increase in facility delivery, PNC coverage in many low-income and middle-income countries (LMICs) remains below 50%.^2^ In many LMICs, observation within a facility for 24 hours after delivery is challenging. This is in part due to pressure from families to leave after an uncomplicated delivery, lack of staffing and infrastructure for inpatient care, facility opening and closing times and a significant proportion of home deliveries.^3 4^

Evidence from LMICs with high newborn mortality rates demonstrates that early, quality PNHVs, within 72 hours after birth, can reduce newborn deaths by between 30%–61% through support for healthy postnatal practices and early identification of danger signs and referral.^5^ Yet high coverage of PNHVs is difficult to achieve in most LMICs, particularly due to limited coverage of CHW cadres.^6^

However, where adequate human resources are made available, evidence demonstrates benefit in identifying and providing risk-stratified PNHVs to mother–baby dyads.^7^ Such an approach would identify and prioritise at-risk mother–baby dyads at the facility and at home for early PNHVs using evidence-informed criteria to identify those at risk of an adverse outcome.^8 9^ Criteria can be clinical (eg, medical conditions and complications) or non-clinical (eg, sociodemographic, household, environmental factors). Using these criteria, health providers categorise mother–baby dyads based on risk and proactively create client-specific care plans.^10^ A limited number of nascent programme experiences have provided initial results and lessons,^11^ buttressed by a review of PNHV approaches that identified the need for ‘specifically targeting high-risk mothers and newborns for PNHVs, rather than using a ‘blanket approach’ that attempts to reach all mothers and newborns’.^12^ Yet the overall field lacks consensus around the need for a risk-stratified PNC approach, and the essential considerations for such an approach. Further, evidence from other fields of medicine has shown that a narrow focus using a risk-stratified approach could lead to unintended negative consequences including missing clients with no identifiable risk factors and potential for stigmatisation.^13 14^

To inform the development and implementation of a risk-stratified PNC approach in LMICs, an iterative scoping literature review to identify risk criteria and an expert consultation were conducted. This paper presents the findings and recommendations from the expert consultation; findings from the scoping review will be published separately.

### Methodology

A team of maternal and newborn health (MNH) experts, selected for their PNC expertise and drawn from academia, implementation partners and donors, were invited for a facilitated virtual expert consultation in April 2021. The consultation aimed to:

1. Review key risk factors, drawn from the literature review, for use at service delivery point (facility, community) to identify at-risk mother–baby dyads.
2. Identify key considerations to prioritise risk factors and operationalise a risk-stratified PNC approach.

A discussion guide was developed in line with the two key objectives, pretested with an MNH expert who was not part of the consultation and used to facilitate the meeting. Discussion questions were high-level to encourage engagement:

1. In your experience, what are the major risk factors, both proximate and distal, that predict poor outcomes in the postnatal period for both mother and baby?
2. What key issues or considerations should be taken into account when selecting risk factors for use in a risk stratification approach in different contexts?

The consultation was held on Zoom for 90 min. Consent was sought from the participants to record the proceedings and use the recordings while ensuring that all participant information was de-identified. An inductive analysis process was used, and data were coded into emerging themes following transcription.

### Findings

Seventeen MNH experts participated in the consultation. The discussion mainly explored key considerations for prioritisation and operationalisation. The findings are presented along the key themes that emerged during the discussion.

### Risk factors identified from the literature review

The risk factors identified from the iterative scoping literature review (box 1) were presented for the experts to reflect on and identify any additional factors based on their research and experience. The scoping review focused on population-based studies and excluded hospital-based studies and therefore the criteria identified were mainly non-clinical rather than the clinical risk factors traditionally used to screen for risk.

Box 1 **Factors associated with poor outcomes for mothers and newborns in the postnatal period (*full list is presented in the scoping review paper*)**

Proximate factors include maternal age (<20, >35), primiparity and grand multiparity, shorter birth intervals, first order/rank neonates, male neonates, birth weight (smaller and larger than average), multiple gestation, previous history of death of child <5 years and lack of or inadequate antenatal care.

Distant factors include low levels of parental education (lower than primary), parental employment (no employment or informal employment), rural residence, low household income, use of solid fuels and lack of clean water.

The risk factors presented have been known to the MNH community for decades. The participants raised the importance of strengthening initiatives that address and eliminate these risk factors in addition to applying them for screening purposes. Additionally, they identified the role of broader, emerging issues such as climate change, conflict, displacement and disease outbreaks in aggravating the proximate and distant risk factors which puts a larger proportion of mother–baby dyads at risk.

### Key considerations for the operationalisation of a risk-stratified PNC approach

#### Framing risk-stratified PNC approaches in the context of universal health coverage

Achieving universal health coverage (UHC) for PNC means providing quality, timely, accessible, equitable services for all mother–baby dyads, regardless of place of birth. Thus, it is critical to understand how a PNC approach that prioritises a subset of mothers and babies contributes to these aims. The journey towards achieving UHC is incremental and equity-focused, creating opportunities for risk-stratified PNC approaches that identify and prioritise those already facing poorer outcomes.

A risk-stratified PNC approach still requires a strengthened health system that can provide optimal PNC services, as the selected quotes in box 2 illustrate. This includes strengthened provider capacity in PNC; adequate supply of essential medicines and equipment; strong referral systems including community follow-up; timely, reliable, quality data for risk screening; functional monitoring systems to assess functionality of the risk-stratified PNC approach and the provision of respectful, dignified care.

Box 2 **Selected quotes from participants on framing risk-stratified approaches within the context of universal health coverage**

We've been wondering whether focused approach and risk-stratified approach for the babies at most risk would be a more efficient way of doing it because our universal approach as you know, has been very challenging. It would be important to discuss this risk-stratified approach but at the same time, you know balancing the universal approach, I think, somehow being able to do both will be important. **Participant 3, F**

If you are looking at this risk factor I go back to the skills. Do they know how to identify this woman who is at risk, do they know how to deal with a woman who is at risk? **Participant 15, F**

There are so many things that’s tied to it [risk screening] like data to screen and to track morbidity and outcomes….and then the women’s experience of care, and often that's forgotten…. **Participant 12, F**

#### Framing risk-stratified early PNHVs in the context of rising coverage of facility delivery

A benefit of the risk-stratified PNC approach is to prioritise limited community-level resources towards early PNHVs for at-risk mother–baby dyads. The rising coverage of facility deliveries and the missed opportunities to provide quality early PNC at facility level raised questions on whether a community-based risk-stratified PNC approach is still relevant and if more emphasis should be placed on quality facility-level PNC.

Despite the rising global coverage of facility delivery, a significant proportion of mothers still deliver at home in many LMICs, and many are discharged before the recommended 24 hours. Again, some categories of at-risk mother–baby dyads such as adolescent mothers or mothers with small and sick newborns will still require PNHVs even with strengthened, quality PNC services at facility level. Box 3 provides select expert quotes that illustrate this point.

Box 3 **Selected quotes from participants on framing early postnatal home visits in the context of rising coverage of facility delivery**

I think, personally, facility delivery is increasing and there are a lot of issues at facility level. I think, ideally, we should focus on improving the quality of services provided to mother and baby at facility level… increasingly I think what we really need is a strategy that addresses quality at the facility. **Participant 1, M**

I think we are seeing more and more women deliver in the facility, but we are not seeing a reduction in [postnatal] mortality due to quality issues. If we could improve the quality of care during childbirth and have those who are at risk stay longer, we may see a return on investment in saving mothers’ and newborns’ lives. **Participant 14, F**

#### Selection of type of risk factors to use in a screening approach

There is value in including non-clinical risk factors in a screening approach. However, the challenges of their operationalisation may be the reason why risk screening approaches have largely used clinical factors. For example, several of the factors identified are difficult to use for rapid screening at service delivery point by a health provider and could create stigma or embarrassment (eg, household income). Some clinical risks can also be challenging to use in rapid screening (eg, body mass index).

A tiered approach that begins with clinical risk factors, which are more acceptable and easier to use, and then includes the non-clinical risks could mitigate this challenge. Alternatively, selecting both clinical and non-clinical risks factors based on ease of use at service delivery level could address the challenge. Box 4 provides select quotes that illustrate this point.

Box 4 **Selected quotes from participants on selection of risk factors for use**

And yes, I do agree that, in addition to the clinical aspects of the risk factor, also looking at the other determinants like socio-economic elements that put a baby at risk, I think, are important also to include. Again, balancing all of this, you know so that it’s programmable—that is the biggest challenge. **Participant 3, F**.

May I suggest start with a clinical approach defined by context… **Participant 13, M**.

I like that idea of a tiered approach because starting with all the factors including the socioeconomic ones can be very difficult, so the suggestion of a tiered approach would work well. **Participant 6, F**.

#### Mitigating negative unintended consequences

Every pregnancy is a high-risk event. Many mothers and babies who develop complications in the postnatal period lack identifiable risk factors, and a risk-stratified approach should also rapidly identify and manage them. Risk-stratified PNC approaches must be nested within PNC strengthening initiatives so that the broader system acts as the safety net that catches those without identifiable risk factors and, thus, do not meet the screening criteria.

As one expert noted, improvements in overall quality and use of PNC by all women, including those not identified as at-risk, have been seen in areas where risk-stratified PNC approaches were used, highlighting the potential of a knock-on effect with implications for strengthening PNC for all women. As illustrated by the selected quotes in box 5, this points towards a potential inherent risk mitigation factor that should be studied further.

Box 5 **Selected quotes from participants on mitigating negative unintended consequences**

Certainly risk stratification is crucial and being able to identify moms and babies, who are more likely to have poor outcomes. I think we also know that sometimes those poor outcomes come from nowhere for both the mother and the baby. I feel like we need to consider also what a dual strategy is so that there’s a specific strategy that deals with the mothers and babies who are more at risk and more likely to have those poor outcomes. And then, a broader based community strategy that can detect those issues that seem to come from nowhere for mothers and babies who don’t appear to have any risk factors, but then subsequently develop significant issues. **Participant 10, F**.

What was found in one study is by initially concentrating on that risk stratification that indeed it led to improvements in PNC numbers, quality and content overall so you know again that kind of speaks to the theory of by concentrating on one aspect all boats rise… **Participant 6, F**.

## Discussion

Timely and quality postnatal care is critical for mothers and newborns. Yet in LMICs, PNC coverage remains stubbornly low^2^ despite increased facility delivery. Prior risk stratification efforts have sought to identify and prioritise at-risk mothers during pregnancy.^9 15^ Yet limited efforts have targeted at-risk mother–baby dyads during the postnatal period,^1 16^ and little global consensus around the need for a risk-stratified PNC approach, and the considerations for such an approach, exists. Given the risk of stigma resulting from labelling mothers as ‘at-risk’, the term ‘targeted PNC’ may be more suitable for real-world application than ‘risk stratification’ and is thus used throughout this discussion.

The expert consultation concluded that concurrent efforts are needed to target coverage of PNC to those most at risk of adverse outcomes, while improving quality of PNC to meet the increasing coverage of facility delivery. Through providing an equity lens to guide systematic identification of those most vulnerable to poor postnatal outcomes, targeted PNC should be considered a contribution—not an alternative—to UHC efforts. PNC approaches targeting those most at-risk of mortality in the postnatal period also contributes to the attainment of the third Sustainable Development Goal.

We suggest that targeted PNC can be advanced in parallel, and as a contribution, to UHC efforts. In the short term, community-based provider cadres must be sufficiently resourced and staffed to allow screening of all mother–baby dyads, adequate counselling on danger signs, timely identification and outreach to at-risk mother–baby dyads and rapid identification and referral for those who later develop complications. In the medium term, universal coverage of PNHVs can only be achieved when CHW-to-household ratios are fully adequate, and transportation is available for CHWs to reach assigned households; this requires advocacy with government to deepen investments in CHWs. Targeted PNHVs would be phased out as an adequate CHW-to-household ratio is reached and blanket PNHV coverage can be achieved. Longer-term investments are needed to address gaps in physical infrastructure and human resources, as well as social challenges that limit use of facility-based services, degrade service quality and discourage longer stays. Further, while ANC coverage is generally higher,^16^ efforts to strengthen coverage and quality of ANC are needed in tandem to improve detection of at-risk mother–baby dyads and encourage continuity of care.

Targeted PNC should be considered and provided in the presence of certain conditions. First, targeted PNC is only appropriate in the context of efforts to strengthen the timing and quality of facility PNC, including pre-discharge PNC, for all mother–baby dyads. This allows for identification and timely service provision for those who develop complications even in the absence of identifiable risk factors. Second, monitoring systems must allow both timely identification of mother–baby dyads meeting established risk criteria, and proactive tracking, identification and resolution of any unintended consequences.

Implementing a targeted PNC approach nested within broader equity-based UHC efforts entails consideration of how limited resources can be most effectively and efficiently targeted to those most likely to benefit. Exploration of several key considerations through robust country learning agendas is needed. First, decisions of which mother–baby dyads should be targeted should be guided by identification of risk factors comprising both clinical and non-clinical predictors of poor outcomes. Evidence-based risk criteria for both facility-based and community-based providers must be determined with consideration of both contextual relevance and feasibility of operationalisation.

Next are considerations of how to operationalise selected evidence-informed clinical and non-clinical risk factors by facility and community providers. The timing of risk identification merits further consideration (ie, some factors may be identifiable during pregnancy, while others manifest only following delivery). Clear and feasible guidance on actions to be taken for mother–baby dyads meeting risk criteria is needed and must be developed with careful consideration of the implications for provider workload and motivation, client flow and facility infrastructure capacity. Given the vulnerability of at-risk mother–baby dyads, particularly those with identified non-clinical risks, efforts to increase accessibility and ensure respectful care are particularly critical elements of broader UHC efforts. Unintended consequences—positive and negative impacts on the health system and on health outcomes—must be assessed, monitored continuously and addressed in consultation with health workers and policymakers. Further, efforts are needed to gather perspectives of mothers, their families and communities to understand the acceptability of a targeted PNC approach and to identify unintended consequences from clients’ perspectives.

Notably, broader efforts are needed to reduce prevalence of underlying clinical and non-clinical risk factors that contribute to poor maternal and newborn outcomes. Mitigating the non-clinical risk factors will require a multisectoral effort beyond the health system.

This consultation has several limitations. The expert consultation invited perspectives of a small number of global and country experts. While the format facilitated robust engagement of experts with deep and diverse expertise in the subject matter, and involvement in strategy and policy from the organisational to global levels, findings represent the perspectives of a small and targeted sample. While care was taken to ensure diversity of experts’ sex, organisation affiliation and country of origin, perspectives of other relevant stakeholders are not represented. Notably, all experts came from a clinical background, which shaped perspectives shared. The discussion explored high-level policy considerations, and did not explore acceptability of targeted PNC from the perspectives of mothers, families or health workers. The discussion did not allow for confidentiality, which could have led to social desirability bias.

## Conclusion

Targeted community-based PNC approaches, nested within broader efforts to strengthen quality PNC services including pre-discharge PNC, could improve outcomes for mother–baby dyads most at-risk of morbidity and mortality during the postnatal period.

## Supplementary Material

Reviewer comments

Author's manuscript

## Data Availability

No data are available.
