# Supplementary material for: On the road to universal coverage of postnatal care: considerations for a targeted postnatal care approach for at-risk mother–baby dyads in low-income and middle-income countries informed by a consultation with global experts
Source: BMJ Open. 2022 Jun 14;12(6):e058408. doi: 10.1136/bmjopen-2021-058408 (PMC9198691; doi:10.1136/bmjopen-2021-058408)
Supplement: Author's manuscript [file bmjopen-2021-058408.draft_revisions.pdf]

BMJ Open is committed to open peer review. As part of this commitment we make the peer review history of every article we publish publicly available.

When an article is published we post the peer reviewers' comments and the authors' responses online. We also post the versions of the paper that were used during peer review. These are the versions that the peer review comments apply to.

The versions of the paper that follow are the versions that were submitted during the peer review process. They are not the versions of record or the final published versions. They should not be cited or distributed as the published version of this manuscript.

BMJ Open is an open access journal and the full, final, typeset and author-corrected version of record of the manuscript is available on our site with no access controls, subscription charges or pay-per-view fees (http://bmjopen.bmj.com).

If you have any questions on BMJ Open's open peer review process please email info.bmjopen@bmj.com

BMJ Open

On the road to universal coverage of postnatal care: Considerations for a targeted approach for at-risk mother-baby dyads informed by an expert consultation

Journal:	*BMJ Open*
Manuscript ID	bmjopen-2021-058408
Article Type:	Original research
Date Submitted by the Author:	17-Oct-2021

Complete List of Authors:	Muriuki, Angela; Save the Children,<br>Yahner, Melanie; Save the Children Federation Inc, Global Health<br>Kiragu, Michael; Adroitz Consultants<br>de Graft-Johnson, Joseph; Save the Children Federation Inc<br>IZULLA, PRESTON; Adroitz Consultants Limited,

Keywords:	Health policy < HEALTH SERVICES ADMINISTRATION & MANAGEMENT, Quality in health care < HEALTH SERVICES ADMINISTRATION & MANAGEMENT, Public health < INFECTIOUS DISEASES, Maternal medicine < OBSTETRICS, Community child health < PAEDIATRICS

SCHOLARONE™
Manuscripts

I, the Submitting Author has the right to grant and does grant on behalf of all authors of the Work (as defined in the below author licence), an exclusive licence and/or a non-exclusive licence for contributions from authors who are: i) UK Crown employees; ii) where BMJ has agreed a CC-BY licence shall apply, and/or iii) in accordance with the terms applicable for US Federal Government officers or employees acting as part of their official duties; on a worldwide, perpetual, irrevocable, royalty-free basis to BMJ Publishing Group Ltd ("BMJ") its licensees and where the relevant Journal is co-owned by BMJ to the co-owners of the Journal, to publish the Work in this journal and any other BMJ products and to exploit all rights, as set out in our licence.

The Submitting Author accepts and understands that any supply made under these terms is made by BMJ to the Submitting Author unless you are acting as an employee on behalf of your employer or a postgraduate student of an affiliated institution which is paying any applicable article publishing charge ("APC") for Open Access articles. Where the Submitting Author wishes to make the Work available on an Open Access basis (and intends to pay the relevant APC), the terms of reuse of such Open Access shall be governed by a Creative Commons licence – details of these licences and which Creative Commons licence will apply to this Work are set out in our licence referred to above.

Other than as permitted in any relevant BMJ Author's Self Archiving Policies, I confirm this Work has not been accepted for publication elsewhere, is not being considered for publication elsewhere and does not duplicate material already published. I confirm all authors consent to publication of this Work and authorise the granting of this licence.

For peer review only

On the road to universal coverage of postnatal care: Considerations for a targeted approach for at-risk mother-baby dyads informed by an expert consultation

Muriuki, Angela[1]
Yahner, Melanie[2]
Kiragu, Michael[3]
De Graft-Johnson, Joseph[2]
Izulla, Preston[3]

[1] Save the Children, Kenya
[2] Save the Children, United States
[3] Adroitz Consultants Limited, Kenya

Correspondence to:
Full name: Dr Angela W Muriuki
Mailing address: Save the Children, East and Southern Africa Regional Office, 2nd Floor ABC Place, Waiyaki Way, Westlands.
P.O Box 19423 - 202, Nairobi, Kenya
Email: amuriuki@savechildren.org
Phone: +254 71 109 00 00

Word count: 3673
References: 27

Abstract:

Introduction: The potential of timely, quality postnatal care (PNC) to reduce maternal and newborn mortality and to advance progress toward Universal Health Coverage (UHC) is well-documented. Yet, in many low- and middle-income countries, coverage of PNC remains low. Risk-stratified approaches can maximize limited resources by targeting mother-baby dyads meeting the evidence-based risk criteria which predict poor postnatal outcomes.

Objectives: To review evidence-based risk criteria for identification of at-risk mother-baby dyads, drawn from a literature review, and to identify key considerations for their use in a risk-stratified PNC approach.

Design/setting/participants: A virtual, semi-structured group discussion was conducted with maternal and newborn health experts on Zoom™. Participants were identified through purposive sampling based on content and context expertise.

For peer review only - http://bmjopen.bmj.com/site/about/guidelines.xhtml

Results: Seventeen experts, (5 male, 12 female), drawn from policymakers, implementing agencies and academia participated and surfaced several key themes. The identified risk factors are well known necessitating accelerated efforts to address underlying drivers of risk. Risk-stratified PNC approaches complement broader UHC efforts by providing an equity lens to identify the most vulnerable mother-baby dyads. However, these should be layered on efforts to strengthen PNC service provision for all mothers and newborns. Risk factors should comprise context relevant, operationalizable, clinical and non-clinical factors. Even with rising coverage of facility delivery, targeted postnatal home visits still complement facility-based PNC.

Conclusion: Risk-stratified PNC efforts must be considered within broader health systems strengthening efforts. Implementation research at the country level is needed understand feasibility and practicality of clinical and non-clinical risk factors and identify unintended consequences.

Article summary

Strengths and limitations of this study

- A major strength of this study is the depth and breadth of expertise of the participants in PNC, each bringing a combination of clinical, research, policy, and implementation skills across multiple low-and-middle-income countries.
- The study explored a critical area of maternal and newborn health where limited evidence exists, highlighting the role of targeted PNC within the current UHC context and its operationalization.
- However, the consultation did not include experts representing Ministries of Health or other government stakeholders as ultimate custodians of a targeted PNC approach.
- In addition, nearly all experts came from a clinical background, which shaped perspectives shared.
- The discussion platform did not allow for confidentiality, which could have led to social desirability bias.

For peer review only - http://bmjopen.bmj.com/site/about/guidelines.xhtml

Introduction

Approximately 66% of maternal deaths and 75% of neonatal deaths occur within the first week after delivery.[1] WHO recommends postnatal care (PNC) at a facility, within 24 hours after birth, regardless of place of birth, observation within a facility for at least 24 hours after delivery, and early postnatal home visits by community health workers (CHWs) to complement facility-based PNC.[2] Efforts to expand coverage of quality, timely, equitable PNC services to all mothers and their newborns directly contribute to the achievement of the 3rd Sustainable Development Goal overall, including to target 3.8, which relates to the attainment of Universal Health Coverage (UHC).[3,4]

Despite an increase in facility delivery, PNC coverage in many low- and middle-income countries (LMICs) remains below 50%.[5] In many LMICs, observation within a facility for the recommended 24 hours after delivery is challenging. This is in part due to pressure from families to leave after an uncomplicated delivery, lack of staffing and infrastructure for inpatient care, facility opening and closing times and a significant proportion of home deliveries.[6,7]

Evidence from LMICs with high newborn mortality rates demonstrates that early, quality postnatal home visits, within 72 hours after birth, can reduce newborn deaths by between 30-61% through support for healthy postnatal practices and early identification of danger signs and referral.[8] Yet, in many LMICs, the CHW cadres responsible for conducting home visits are under-resourced and inadequately trained, supervised, and remunerated.[9] Where high coverage of postnatal home visits is difficult to achieve due to these challenges, as in most LMICs, evidence demonstrates benefit in identifying and providing postnatal home visits to mother – baby dyads who face a higher risk of poor outcomes.[10]

Risk-stratified PNC approaches aim to identify and prioritize at-risk mother-baby dyads at the facility and at home for early postnatal home visits using evidence-informed criteria to identify those at risk of an adverse outcome.[11,12] Using these criteria, health providers categorize mother-baby dyads based on risk and proactively create client-specific care plans.[13] While criteria can be clinical (e.g., medical conditions and complications) or nonclinical (e.g., sociodemographic, household, environmental factors), most risk stratification efforts have used clinical criteria, and most have focused on risk identification in pregnancy.[14,15]

Evidence from other fields of medicine has shown that a narrow focus using a risk-stratified approach could lead to unintended negative consequences including missing clients with no identifiable risk factors and could create room for stigmatization.[16,17] Thus, it is crucial to layer

For peer review only - http://bmjopen.bmj.com/site/about/guidelines.xhtml

the approach on, and integrate it into, existing quality improvement and UHC efforts aiming to provide quality, timely PNC for all mothers and their newborns.

To inform the development and implementation of this approach, an iterative scoping literature review to identify risk criteria for use and an expert consultation were conducted. This paper presents the findings and recommendations from the expert consultation; findings from the scoping review will be published separately.

Methodology

To gain a better understanding of the identification and use of risk criteria in the provision of PNC services, a qualitative approach using a semi-structured virtual group discussion was used. A team of maternal and newborn health (MNH) experts, drawn from academia, implementation partners and donors, were invited for a facilitated virtual expert consultation in April 2021. Participants were selected through purposive sampling based on their ability to provide in-depth and detailed information on the subject matter (PNC) and context (LMICs) due to their experience and expertise. The participants were contacted via email. The consultation aimed to:

1. Review key risk factors, drawn from the literature review, for use at service delivery points (facility, community) to identify at-risk mother-baby dyads.
2. Identify key considerations to prioritize risk factors and operationalize a risk-stratified PNC approach.

A discussion guide was developed in line with the two key objectives, pretested with an MNH expert who was not part of the consultation and used to facilitate the meeting. The consultation was held on Zoom™ for ninety minutes and facilitated by two trained researchers who were not known to the participants. In addition to the researchers and participants, there were two observers, one note-taker and one person managing the virtual meeting logistics.

Since the discussion aimed to seek the experts' opinions on a subject matter rather than information on the experts themselves, it was deemed to be non-human subjects research and therefore did not require ethical approval. However, verbal consent was sought from the participants to record the proceedings and use the recordings while ensuring that all participant information was de-identified. Experts were given the option to opt out of the recorded session and those who participated provided consent.

The initial emerging themes were identified during the virtual discussion and summarised at the end of the consultation by the facilitators and one of the observers. The themes were shared with the experts via email to elicit any additional reflections. Data saturation was not

For peer review only - http://bmjopen.bmj.com/site/about/guidelines.xhtml

discussed. The audio transcript generated by Zoom™ was used for data analysis with the audio recording used to correct any errors in the transcript. A deductive approach was used to code the data along the initial themes and to assess for any new emerging themes after which thematic analysis was conducted. Further exploration of the initial themes and new emerging themes was conducted by the full research team.

Findings

Of the twenty-one invited MNH experts, seventeen participated in the consultation. Five of the participants were male. For two of the non-participants, the reason given was unavailability during the identified time slot due to prior commitments while one was on leave and was not able to participate. Two did not respond to the invite. The findings are presented along the key themes that emerged from the data.

1. Reflection on risk factors identified from the literature review

The risk factors identified from the iterative, scoping literature review were presented for the experts to reflect on and to identify additional factors based on their research and experience. The scoping review focused on population-based studies and excluded hospital-based studies and therefore the criteria identified were non-clinical rather than the clinical risk factors traditionally used to screen for risk.

An adapted version of the Mosley and Chen framework was used for the scoping review.[18] In the framework, distant factors are the broader socioeconomic factors at individual, household, or community level such as education, wealth status and residence. These act through the proximate causes that are primarily bio-behavioural factors related to the mother and/or neonate such as maternal age, birthweight and utilization of health services that are more directly linked to observed clinical manifestations such as infection or bleeding which led to death.

Textbox 1: Factors associated with poor outcomes for mothers and newborns in the postnatal period

Proximate factors include maternal age (<20, >35), primiparity and grand multiparity, shorter birth intervals, first order/rank neonates, male neonates, birth weight (smaller and larger than average), multiple gestation, previous history of death of child aged less than 5 years, and lack of or inadequate antenatal care

Distant factors include low levels of parental education (lower than primary level education), parental employment (no employment or informal employment), rural residence, low household income, use of solid fuels and lack of clean water

For peer review only - http://bmjopen.bmj.com/site/about/guidelines.xhtml

According to the experts, the risk factors presented have been known to the MNH community for decades, yet they still played a significant role in influencing maternal and neonatal outcomes in the postnatal period. This raised the importance of expanding initiatives that address and eliminate these risk factors, such as promoting female education, economic empowerment and addressing factors that lead to early childbearing including child marriage, in addition to applying them for screening purposes.

> *'These require generic interventions so some of the interventions and approaches for those risk drivers are not restricted or limited to the specific risk periods that we are taught, and we have spoken about. There are things that can be part of broad packages of care and just ensuring that you have delivery platforms that can address them.'* ***Participant 13, M.***

Additionally, they identified the role of broader, emerging issues such as climate change, conflict, displacement, and disease outbreaks and their resultant effect in aggravating the proximate and distant risk factors which pushes a larger proportion of mother-baby dyads into the risk categories.

> *'Conflict is one that jumps out to me. Forty percent of the current global burden of maternal newborn adverse outcomes, from our conservative estimation, are related conflicts settings including populations on the move. The risks [in these settings] are very different to the way we look at the those in stable circumstances.'* ***Participant 13, M.***

2. Key considerations for the prioritization of risk factors and operationalization of a risk-stratified PNC approach

a) Framing risk-stratified PNC approaches in the context of universal health coverage

Achieving UHC for PNC means providing quality, timely, accessible, equitable services for all mother-baby dyads, regardless of place of birth. Thus, it is critical to understand how a PNC approach that prioritises a sub-set of mothers and babies contributes to—or detracts from—these aims. There were mixed reactions from the participants on the role of the risk-stratified PNC approaches within the context of achieving PNC coverage for all.

> *'We've been wondering whether focused approach and risk-stratified approach for the babies at most risk would be a more efficient way of doing it because our universal approach as you know, has been very challenging. It would be important to discuss*

For peer review only - http://bmjopen.bmj.com/site/about/guidelines.xhtml

this risk stratified approach but at the same time, you know balancing the universal approach, I think, somehow being able to do both will be important.' ***Participant 3, F.***

However, the experts agreed that the journey towards achieving UHC is incremental and equity-focused, creating opportunities for risk-stratified PNC approaches that identify and prioritise those already facing poorer outcomes.

A risk-stratified PNC approach still relies on a strengthened health system that can provide optimal PNC services for all mothers and their newborns. According to participants, a risk-stratified approach would require strengthened provider capacity in PNC; adequate supply of essential medicines and equipment; strong referral systems including community follow-up; timely, reliable, quality data for risk screening; functional monitoring systems to assess functionality of the risk-stratified PNC approach and the provision of respectful, dignified care.

'If you are looking at this risk factor I go back to the skills. Do they know how to identify this woman who is at risk, do they know how to deal with a woman who is at risk?' ***Participant 15, F.***

'There are so many things that's tied to it [risk screening] like data to screen and to track morbidity and outcomes….and then the women's experience of care, and often that's forgotten…' ***Participant 12, F.***

I was thinking, one of the things that needs to be taken into consideration is what exists in terms of the community health system…so that probably is going to guide us in what can be done in terms of approaches.' ***Participant 6, F***.

One expert emphasised the need for robust data for accurate screening and outcome tracking which could pose a significant challenge to implementation in many LMICs due to weak data systems. The expert noted that data availability also informs the refinement of the approach through a continuous review of those identified as at-risk and those with no identifiable risk factors who later develop complications.

b) Framing risk-stratified early postnatal home visits in the context of rising coverage of facility delivery

A benefit of the risk-stratified PNC approach is to prioritise limited community-level resources towards early postnatal home visits for at-risk mother-baby dyads. The rising coverage of facility deliveries and the missed opportunities for providing quality early PNC at facility level led to questions on whether a community-based risk-stratified PNC approach was still relevant and if more emphasis ought to be placed on quality PNC at facility level.

For peer review only - http://bmjopen.bmj.com/site/about/guidelines.xhtml

Despite the rising global coverage of facility delivery, participants noted that a significant proportion of mothers still deliver at home in many LMICs, and many of those who deliver in a facility are discharged before the recommended 24 hours. Again, some categories of at-risk mother-baby dyads such as adolescent mothers or mothers with small and sick newborns will still require postnatal home visits even with strengthened, quality PNC services at facility level.

> ‘*I think, personally, facility delivery is increasing and there are a lot of issues at facility level. I think, ideally, we should focus on improving the quality of services provided to mother and baby at facility level… increasingly I think what we really need is a strategy that addresses quality at the facility.*’ ***Participant 1, M.***

> *‘‘I think we are seeing more and more women deliver in the facility, but we are not seeing a reduction in [postnatal] mortality due to quality issues. If we could improve the quality of care during childbirth and have those who are at risk stay longer, we may see a return on investment in saving mothers and newborns lives.’* ***Participant 14, F.***

c) Selection of risk factors and timing of screening

According to the experts, there is value in using both clinical and non-clinical risk factors in a screening approach. They, however, noted that the challenges of their operationalization may be why risk screening approaches have used clinical factors. For example, several of the factors identified are difficult to use for rapid screening at service delivery point by a health provider and could create stigma or embarrassment (e.g., household income). Some clinical risks can also be challenging to use in rapid screening (e.g., body mass index). Selecting both clinical and non-clinical risks factors based on feasibility of use at service delivery level could address the challenge.

> *‘And yes, I do agree that, in addition to the clinical aspects of the risk factor, also looking at the other determinants like socio-economic elements that put a baby at risk, I think, are important also to include. Again, balancing all of this, you know so that it's programmable—that is the biggest challenge.’* ***Participant 3, F.***

> *‘May I suggest start with a clinical approach defined by context…’* ***Participant 13, M.***

The targeted PNC approach proposes the assessment of risk after birth. However, some of the identified risk factors are either already present or manifest during pregnancy. Therefore, experts emphasised on the importance of conducting the screening during the first contact in pregnancy to identify and, where possible, address these risk factors as early as possible to improve outcomes through pregnancy and into the postpartum period.

For peer review only - http://bmjopen.bmj.com/site/about/guidelines.xhtml

d) Mitigating negative unintended consequences

The participants emphasised that every pregnancy is a high-risk event and many mothers and babies who develop complications in the postnatal period lack identifiable risk factors. Therefore, a risk-stratified approach approaches must be nested within PNC strengthening initiatives so that the broader system acts as the safety net that catches those without identifiable risk factors and, thus, do not meet the screening criteria.

> *'Certainly, risk stratification is crucial and being able to identify moms and babies, who are more likely to have poor outcomes. I think we also know that sometimes those poor outcomes come from nowhere for both the mother and the baby. I feel like we need to consider also what a dual strategy is so that there is a specific strategy that deals with the mothers and babies who are more at risk and more likely to have those poor outcomes. And then, a broader based community strategy that can detect those issues that seem to come from nowhere for mothers and babies who do not appear to have any risk factors, but then subsequently develop significant issues.'* **Participant 10, F.**

As one expert noted, improvements in overall quality and use of PNC by all women, including those not identified as at-risk, have been seen in areas where risk-stratified PNC approaches were used, highlighting the potential of a knock-on effect with implications for strengthening PNC for all women. This points towards a potential inherent risk mitigation factor that should be studied further.

> *'What was found in one study is by initially concentrating on that risk stratification that indeed it led to improvements in PNC numbers, quality, and content overall, so you know again that kind of speaks to the theory of by concentrating on one aspect all boats rise…'* **Participant 6, F.**

Discussion

Timely and quality postnatal care is increasingly recognized as critical for mothers and their newborns. Yet in many LMICs, PNC coverage remains stubbornly low despite increased facility delivery. While prior risk stratification efforts have sought to identify and prioritize at-risk mothers during pregnancy,[19,20] limited efforts have targeted at-risk mother-baby dyads during the postnatal period.[21] The findings of this expert consultation underscore the potential contribution of identifying at-risk mother-baby dyads based on clinical and non-clinical risk criteria to broader UHC efforts and raise key considerations for the operationalization of risk-stratified PNC approaches.

For peer review only - http://bmjopen.bmj.com/site/about/guidelines.xhtml

Given the risk of stigma resulting from labelling mothers and newborns as 'at-risk,' the term 'targeted PNC' may be more suitable for real-world application than 'risk stratified PNC' and is thus used throughout this discussion.

The expert consultation concluded that concurrent efforts are needed to target coverage of PNC to those most at-risk of adverse outcomes, while improving quality of and access to PNC to meet the increasing coverage of facility delivery. Through providing an equity lens to guide systematic identification of those most vulnerable to poor postnatal outcomes, targeted PNC should be considered a contribution—not an alternative—to UHC efforts.

The expert consultation concluded that targeted PNC should be considered and provided in the presence of certain conditions. First, targeted PNC is only appropriate in the context of efforts to strengthen the timing and quality of facility PNC, including pre-discharge PNC, for all mother-baby dyads. This allows for identification and timely service provision for those who develop complications in the absence of identifiable risk factors. Community-based provider cadres must be sufficiently resourced (sufficient numbers to achieve population coverage) and supported (through training and supportive supervision)[22] to allow for adequate counselling for caregivers on identification of dangers signs and immediate care-seeking, timely identification and outreach to at-risk mother-baby dyads who later develop complications and rapid referral. Second, monitoring systems, which often include limited data related to PNC,[23] must be strengthened allow both timely identification of mother-baby dyads meeting established risk criteria, and proactive tracking, identification, and resolution of any unintended consequences.

Implementing a targeted PNC approach nested within broader equity-based UHC efforts entails consideration of how limited resources can be most effectively and efficiently targeted to those most likely to benefit. Exploration of several key considerations through robust country learning agendas is needed. First, decisions of which mother-baby dyads should be targeted should be guided by identification of risk factors comprising both clinical and non-clinical predictors of poor outcomes. While clinical risk factors may more feasibly identifiable, evidence demonstrates that non-clinical factors may be equally important to consider; for example, McCarthy et al found that mothers with higher household wealth were more likely to receive a postnatal home visit than women in poorer households.[24] Evidence-based risk criteria for both facility- and community-based providers must be determined with consideration of both contextual relevance and feasibility of operationalization.

Next are considerations of how to operationalize selected evidence-informed clinical and non-clinical risk factors by facility and community providers. The timing of risk identification merits further consideration (i.e., some factors may be identifiable during pregnancy, while others

For peer review only - http://bmjopen.bmj.com/site/about/guidelines.xhtml

manifest only following delivery). Clear and feasible guidance on actions to be taken for mother-baby dyads meeting risk criteria is needed and must be developed with careful consideration of the implications for provider workload and motivation, client flow, and facility infrastructure capacity. In addition to tailored criteria, the identification mechanisms and associated actions for mother-baby dyads identified to meet risk criteria will need to be differentiated for facility- and community-based providers.

Increasingly, the role of service quality, including respectful treatment at facilities, is recognised as a factor to initiation and continuation of service use. [25,26] Given the vulnerability of at-risk mother-baby dyads, particularly those with identified non-clinical risks (e.g., adolescent mothers, those with low socio-economic status), efforts to increase accessibility and ensure respectful care at all levels of the health system are particularly critical elements of broader UHC efforts. Unintended consequences of a targeted PNC approach—positive and negative impacts on the health system and on health outcomes—must be assessed, monitored continuously, and addressed.

Notably, broader efforts are needed to reduce prevalence of underlying clinical and non-clinical risk factors that contribute to poor maternal and newborn outcomes.[27] Mitigating the non-clinical risk factors will require a multi-sectoral effort beyond the health system. Further efforts are needed to deepen understanding of how issues such as climate change, conflict, displacement, and disease outbreaks may confound the proximate and distant risk factors.

This consultation has several limitations. The expert consultation invited perspectives of a small number of global and country experts. While care was taken to ensure diversity of experts' sex, organisation affiliation, and country of origin, perspectives of other relevant stakeholders are not represented. Importantly, no experts represented Ministries of Health or other government stakeholders as ultimate custodians of a targeted PNC approach. In addition, nearly all experts came from a clinical background, which shaped perspectives shared. The discussion did not allow for confidentiality, which could have led to social desirability bias.

Conclusion

Targeted community-based PNC approaches, nestled within broader efforts to strengthen quality PNC services including pre-discharge PNC, could improve outcomes for mother-baby dyads most at-risk of morbidity and mortality during the postnatal period. This paper makes the following key points:

- Efforts are needed to increase coverage of quality, timely PNC to mother-baby dyads most at risk of poor health outcomes in the postnatal period while advancing progress

For peer review only - http://bmjopen.bmj.com/site/about/guidelines.xhtml

toward universal coverage of quality PNC. A targeted PNC approach is one way of achieving this.

- Targeted PNC approaches should be considered in tandem with and layered on complementary efforts aiming to strengthen the coverage, timing, and quality of facility PNC for all mother-baby dyads rather than as stand-alone interventions. This allows for timely identification and provision of care or referral for mother-baby dyads who develop complications without identifiable risk factors.
- Evidence-based clinical and non-clinical factors for use to assess risk should be selected based on key considerations including application to the context and feasibility of operationalization at the targeted service delivery point – facility and/or community.
- Context specific evidence is required to deepen insights into the feasibility and operationalizability of clinical and non-clinical risk factors in a real-world setting; capacity of the health system to support a targeted PNC approach while offering quality, timely PNC services for all mothers and their babies; and unintended consequences (both positive and negative) of a targeted PNC approach.

Author contributions

The authors confirm contribution as follows:

AM contributed to the design and implementation of the expert consultation, conceptualization, secondary data analysis and manuscript writing. AM is a clinician, researcher, and a public health expert with 15 years' experience in maternal, newborn and adolescent health.

MY contributed to the design and implementation of the expert consultation, conceptualization, and manuscript writing. MY is a public health expert and researcher with expertise in adolescent and youth health including postnatal care for first time adolescent and young mothers.

MK contributed to the design and implementation of the expert consultation (including as co-facilitator for the virtual group discussion), data analysis, and critical review and feedback. MK is a clinician, public health expert and researcher with 16 years' experience in research, evaluation, and health policy.

JJ contributed to the design and implementation of the expert consultation, conceptualization of the manuscript and critical review and feedback. JJ is a clinician, public health expert and researcher with over 35 years' experience in maternal and newborn health.

For peer review only - http://bmjopen.bmj.com/site/about/guidelines.xhtml

PI contributed to the design and implementation of the expert consultation (including as co-facilitator for the virtual group discussion), data analysis, and critical review and feedback. PI is a clinician, public health expert and researcher.

Acknowledgements

The authors acknowledge the administrative and logistical support provided by Rudo Kashiri and Amy Mangieri from Save the Children throughout the planning and implementation process of the expert consultation.

Funding statement

This work was supported by the Bill & Melinda Gates Foundation grant number [OPP1199380]. The findings and conclusions contained within are those of the authors and do not necessarily reflect positions or policies of the Bill & Melinda Gates Foundation.

Patient involvement

No patients or members of the public were involved in the design, or conduct, or reporting, or dissemination plans of the research.

Conflicts of Interest

Having read and understood the BMJ policy on declaration of interests, the authors have no conflict of interest to declare.

Licence

The Corresponding Author has the right to grant on behalf of all authors and does grant on behalf of all authors, an exclusive licence (or non-exclusive for government employees) on a worldwide basis to the BMJ Publishing Group Ltd ("BMJ"), and its Licensees to permit this article (if accepted) to be published in The BMJ's editions and any other BMJ products and to exploit all subsidiary rights, as set out in The BMJ's licence.

For peer review only

For peer review only - http://bmjopen.bmj.com/site/about/guidelines.xhtml

393 **References**

[1] World Health Organization. Postnatal care of the mother and newborn [Internet]. Geneva; 2013. Available from: http://apps.who.int/iris/bitstream/10665/97603/1/9789241506649_eng.pdf.

[2] World Health Organization. Postnatal care of the mother and newborn [Internet]. Geneva; 2013. Available from: http://apps.who.int/iris/bitstream/10665/97603/1/9789241506649_eng.pdf.

[3]United Nations. Sustainable Development Goals [Internet]. 2016 [Available from: https:// sustainabledevelopment.un.org/topics/sustainabledevelopmentgoals.

[4] Sacks E, Langlois ÉV. Postnatal care: increasing coverage, equity, and quality. Lancet Glob Health. 2016 Jul;4(7):e442-3. doi: 10.1016/S2214-109X(16)30092-4. Epub 2016 May 13. PMID: 27185467.

[5] Victora, CG, Requejo, JH, Barros, AJD, Berman, P, Bhutta, Z, Boerma T. Countdown to 2015: a decade of tracking progress for maternal, newborn, and child survival. Lancet, The. 2016;387(10032):1049–59.

[6] Bashar Mda, Aggarwal A, Pilania M. Factors causing early maternity discharge of uncomplicated normal delivery cases at government health facilities in Northern India: An exploratory study. J Educ Health Promot [Internet]. 2020;9(1):198. Available from: http://www.jehp.net/text.asp?2020/9/1/198/293940

[7] Campbell OMR, Cegolon L, Macleod D, Benova L. Length of Stay After Childbirth in 92 Countries and Associated Factors in 30 Low- and Middle-Income Countries: Compilation of Reported Data and a Cross-sectional Analysis from Nationally Representative Surveys. Myers JE, editor. PLOS Med [Internet]. 2016 Mar 8;13(3): e1001972. Available from: https://dx.plos.org/10.1371/journal.pmed.1001972

[8] Gogia S, Sachdev HS. Home visits by community health workers to prevent neonatal deaths in developing countries: a systematic review. Bull World Health Organ [Internet]. 2010 Sep 1;88(9):658-666B. Available from: http://www.who.int/bulletin/volumes/88/9/09-069369.pdf

[9] Scott, K., Beckham, S.W., Gross, M. et al. What do we know about community-based health worker programs? A systematic review of existing reviews on community health workers. Hum Resour Health 16, 39. 2018. https://doi.org/10.1186/s12960-018-0304-x

[10] McPherson R, Hodgins S. Postnatal home visitation: Lessons from country programs operating at scale. J Glob Health [Internet]. 2018 Jun;8(1). Available from: http://jogh.org/documents/issue201801/jogh-08-010422.pdf

[11] World Health Organization (WHO). Risk approach for maternal and child health care. In: World Health Forum. 1981. p. 413–22.

[12] Ochejele S, Ijiko E, Obulu M. Assessment of the risk approach in the reduction of maternal mortality in north-central Nigeria. J West African Coll Surg [Internet]. 2011;1(2):76–85. Available from: http://www.ncbi.nlm.nih.gov/pubmed/25452955%0Ahttp://www.pubmedcentral.nih.gov/articlerender.fcgi?artid=PMC4170261

[13] Mora J, Iturralde MD, Prieto L, Domingo C, Gagnon M-P, Martínez-Carazo C, et al. Key aspects related to implementation of risk stratification in health care systems-the ASSEHS study. BMC Health

For peer review only

Serv Res [Internet]. 2017 Dec 5;17(1):331. Available from: http://bmchealthservres.biomedcentral.com/articles/10.1186/s12913-017-2275-3

[14] Rajbanshi S, Norhayati MN, Nik Hazlina NH. High-risk pregnancies and their association with severe maternal morbidity in Nepal: A prospective cohort study. PLoS ONE 15(12): e0244072. 2020 https://doi.org/10.1371/journal.pone.0244072

[15] Patel, A., Prakash, A.A., Pusdekar, Y.V. et al. Detection and risk stratification of women at high risk of preterm birth in rural communities near Nagpur, India. BMC Pregnancy Childbirth 17, 311. 2017 https://doi.org/10.1186/s12884-017-1504-4

[16] Garg A, Boynton-Jarrett R, Dworkin PH. Avoiding the unintended consequences of screening for social determinants of health. JAMA - J Am Med Assoc. 2016;316(8):813–4.

[17] Feudtner C, Schall T, Nathanson P, Berry J. Ethical Framework for Risk Stratification and Mitigation Programs for Children With Medical Complexity. Pediatrics [Internet]. 2018 Mar 1;141(Supplement 3): S250–8. Available from: http://pediatrics.aappublications.org/lookup/doi/10.1542/peds.2017-1284J

[18] Mosley,W,H and Chen, L C. An analytical framework for the study of child survival in developing countries. Bull World Health Organ. 2003;81(2):140–5.

[19] Patel A, Prakash AA, Pusdekar Y V., Kulkarni H, Hibberd P. Detection, and risk stratification of women at high risk of preterm birth in rural communities near Nagpur, India. BMC Pregnancy Childbirth. 2017;17(1).

[20] Ochejele S, Ijiko E, Obulu M. Assessment of the risk approach in the reduction of maternal mortality in north-central Nigeria. J West African Coll Surg [Internet]. 2011;1(2):76–85. Available from: http://www.ncbi.nlm.nih.gov/pubmed/25452955%0Ahttp://www.pubmedcentral.nih.gov/articlerender.fcgi?artid=PMC4170261

[21] World Health Organization. Postnatal care of the mother and newborn [Internet]. Geneva; 2013. Available from: http://apps.who.int/iris/bitstream/10665/97603/1/9789241506649_eng.pdf.

[22] WHO. The World Health Report 2008. Primary Health Care - now more than ever, vol. 26. Geneva: World Health Organization, 2008. Available from: http://www.who.int/whr/2008/whr08_en.pdf

[23] Neal, S., Channon, A.A., Carter, S. et al. Universal health care and equity: evidence of maternal health based on an analysis of Demographic and Household Survey data. Int J Equity Health 14, 56 2015. https://doi.org/10.1186/s12939-015-0184-9

[24] McCarthy KJ, Braganza S, Fiori K, Gbeleou C, Kpakpo V, Lopez A, et al. Identifying inequities in maternal and child health through risk stratification to inform health systems strengthening in Northern Togo. PLoS ONE 12(3): e0173445. 2017. https://doi.org/10.1371/journal.pone.0173445

[25] Bohren MA, Vogel JP, Hunter EC, et al. The mistreatment of women during childbirth in health facilities globally: a mixed-methods systematic review. PLoS Med 2015; 12: e1001847

[26] Asefa A. Unveiling respectful maternity care as a way to address global inequities in maternal health. BMJ Glob Health. 2021;6(1):e003559. doi:10.1136/bmjgh-2020-003559

[27] Batist J. An intersectional analysis of maternal mortality in Sub-Saharan Africa: a human rights issue. J Glob Health. 2019;9(1):010320. doi:10.7189/jogh.09.010320

For peer review only - http://bmjopen.bmj.com/site/about/guidelines.xhtml

COREQ (COnsolidated criteria for REporting Qualitative research) Checklist

A checklist of items that should be included in reports of qualitative research. You must report the page number in your manuscript where you consider each of the items listed in this checklist. If you have not included this information, either revise your manuscript accordingly before submitting or note N/A.

Topic	Item No.	Guide Questions/Description	Reported on Page No.
**Domain 1: Research team and reflexivity**			
Personal characteristics			
Interviewer/facilitator	1	Which author/s conducted the interview or focus group?	
Credentials	2	What were the researcher's credentials? E.g. PhD, MD	
Occupation	3	What was their occupation at the time of the study?	
Gender	4	Was the researcher male or female?	
Experience and training	5	What experience or training did the researcher have?	
Relationship with participants			
Relationship established	6	Was a relationship established prior to study commencement?	
Participant knowledge of the interviewer	7	What did the participants know about the researcher? e.g. personal goals, reasons for doing the research	
Interviewer characteristics	8	What characteristics were reported about the inter viewer/facilitator? e.g. Bias, assumptions, reasons and interests in the research topic	
**Domain 2: Study design**			
Theoretical framework			
Methodological orientation and Theory	9	What methodological orientation was stated to underpin the study? e.g. grounded theory, discourse analysis, ethnography, phenomenology, content analysis	
Participant selection			
Sampling	10	How were participants selected? e.g. purposive, convenience, consecutive, snowball	
Method of approach	11	How were participants approached? e.g. face-to-face, telephone, mail, email	
Sample size	12	How many participants were in the study?	
Non-participation	13	How many people refused to participate or dropped out? Reasons?	
Setting			
Setting of data collection	14	Where was the data collected? e.g. home, clinic, workplace	
Presence of non-participants	15	Was anyone else present besides the participants and researchers?	
Description of sample	16	What are the important characteristics of the sample? e.g. demographic data, date	
Data collection			
Interview guide	17	Were questions, prompts, guides provided by the authors? Was it pilot tested?	
Repeat interviews	18	Were repeat inter views carried out? If yes, how many?	
Audio/visual recording	19	Did the research use audio or visual recording to collect the data?	
Field notes	20	Were field notes made during and/or after the inter view or focus group?	
Duration	21	What was the duration of the inter views or focus group?	
Data saturation	22	Was data saturation discussed?	
Transcripts returned	23	Were transcripts returned to participants for comment and/or	

For peer review only

Topic	Item No.	Guide Questions/Description	Reported on Page No.
		correction?	
**Domain 3: analysis and findings**			
Data analysis			
Number of data coders	24	How many data coders coded the data?	
Description of the coding tree	25	Did authors provide a description of the coding tree?	
Derivation of themes	26	Were themes identified in advance or derived from the data?	
Software	27	What software, if applicable, was used to manage the data?	
Participant checking	28	Did participants provide feedback on the findings?	
Reporting			
Quotations presented	29	Were participant quotations presented to illustrate the themes/findings? Was each quotation identified? e.g. participant number	
Data and findings consistent	30	Was there consistency between the data presented and the findings?	
Clarity of major themes	31	Were major themes clearly presented in the findings?	
Clarity of minor themes	32	Is there a description of diverse cases or discussion of minor themes?	

Developed from: Tong A, Sainsbury P, Craig J. Consolidated criteria for reporting qualitative research (COREQ): a 32-item checklist for interviews and focus groups. *International Journal for Quality in Health Care*. 2007. Volume 19, Number 6: pp. 349 – 357

Once you have completed this checklist, please save a copy and upload it as part of your submission. DO NOT include this checklist as part of the main manuscript document. It must be uploaded as a separate file.

For peer review only

On the road to universal coverage of postnatal care: Considerations for a targeted postnatal care approach for at-risk mother-baby dyads in low- and middle-income countries informed by a consultation with global experts

Journal:	*BMJ Open*
Manuscript ID	bmjopen-2021-058408.R1
Article Type:	Original research
Date Submitted by the Author:	02-Mar-2022
Complete List of Authors:	Muriuki, Angela; Save the Children,<br>Yahner, Melanie; Save the Children Federation Inc, Global Health<br>Kiragu, Michael; Adroitz Consultants<br>de Graft-Johnson, Joseph; Save the Children Federation Inc<br>IZULLA, PRESTON; Adroitz Consultants Limited,
**Primary Subject Heading**:	Global health
Secondary Subject Heading:	Health policy, Obstetrics and gynaecology
Keywords:	Health policy < HEALTH SERVICES ADMINISTRATION & MANAGEMENT, Quality in health care < HEALTH SERVICES ADMINISTRATION & MANAGEMENT, Public health < INFECTIOUS DISEASES, Maternal medicine < OBSTETRICS, Community child health < PAEDIATRICS

SCHOLARONE™
Manuscripts

I, the Submitting Author has the right to grant and does grant on behalf of all authors of the Work (as defined in the below author licence), an exclusive licence and/or a non-exclusive licence for contributions from authors who are: i) UK Crown employees; ii) where BMJ has agreed a CC-BY licence shall apply, and/or iii) in accordance with the terms applicable for US Federal Government officers or employees acting as part of their official duties; on a worldwide, perpetual, irrevocable, royalty-free basis to BMJ Publishing Group Ltd ("BMJ") its licensees and where the relevant Journal is co-owned by BMJ to the co-owners of the Journal, to publish the Work in this journal and any other BMJ products and to exploit all rights, as set out in our licence.

The Submitting Author accepts and understands that any supply made under these terms is made by BMJ to the Submitting Author unless you are acting as an employee on behalf of your employer or a postgraduate student of an affiliated institution which is paying any applicable article publishing charge ("APC") for Open Access articles. Where the Submitting Author wishes to make the Work available on an Open Access basis (and intends to pay the relevant APC), the terms of reuse of such Open Access shall be governed by a Creative Commons licence – details of these licences and which Creative Commons licence will apply to this Work are set out in our licence referred to above.

Other than as permitted in any relevant BMJ Author's Self Archiving Policies, I confirm this Work has not been accepted for publication elsewhere, is not being considered for publication elsewhere and does not duplicate material already published. I confirm all authors consent to publication of this Work and authorise the granting of this licence.

For peer review only

Analysis

On the road to universal coverage of postnatal care: Considerations for a targeted postnatal care approach for at-risk mother-baby dyads in low- and middle-income countries informed by a consultation with global experts

Muriuki, Angela[1]
Yahner, Melanie[2]
Kiragu, Michael[3]
De Graft-Johnson, Joseph[2]
Izulla, Preston[3]

[1] Formerly with Save the Children, Kenya (now independent consultant)
[2] Save the Children, United States
[3] Adroitz Consultants Limited, Kenya

Correspondence to:
Full name: Melanie Yahner
Mailing address: Save the Children, US, 501 Kings Highway East, Fairfield, Connecticut US
Email: myahner@savechildren.org
Phone: +1 475 999 3011

Word count: 2,289
References: 20

KEY MESSAGES

- Efforts are needed to increase coverage of quality, timely postnatal care (PNC) to mother-baby dyads most at risk of poor health outcomes in the postnatal period while advancing progress toward universal coverage of quality PNC. A targeted PNC approach is one way of achieving this.
- Targeted community-based PNC approaches must be considered in tandem with and layered on complementary efforts aiming to strengthen the coverage, timing, and quality of facility PNC for all mother-baby dyads rather than as stand-alone

For peer review only

interventions. This allows for timely identification and provision of care or referral for mother-baby dyads who develop complications without identifiable risk factors.

- Evidence-based clinical and non-clinical factors for use to assess risk should be selected based on key considerations including application to the context and feasibility of operationalization at the targeted service delivery point – facility and/or community.

- Context-specific evidence is required to deepen insights into the feasibility and operationalizability of clinical and non-clinical risk factors in a real-world setting; capacity of the health system to support a targeted PNC approach while offering quality, timely PNC services for all mothers and their babies; and unintended consequences (both positive and negative) of a targeted PNC approach.

Contributors and sources

The authors confirm contribution as follows:

AM contributed to the design and implementation of the expert consultation, conceptualization, secondary data analysis and manuscript writing. AM is a clinician, researcher and a public health expert with 15 years' experience in maternal, newborn and adolescent health.

MY contributed to the design and implementation of the expert consultation, conceptualization, and manuscript writing. MY is a public health expert and researcher with expertise in adolescent and youth health including postnatal care for first time adolescent and young mothers.

MK contributed to the design and implementation of the expert consultative workshop, data analysis, and critical review and feedback. MK is a clinician, public health expert and researcher with 16 years' experience in research, evaluation and health policy.

JJ contributed to the design and implementation of the expert consultation, conceptualization of the manuscript and critical review and feedback. JJ is a clinician, public health expert and researcher with over 35 years' experience in maternal and newborn health.

PI contributed to the design and implementation of the expert consultative workshop, data analysis, and critical review and feedback. PI is a clinician, public health expert and researcher.

For peer review only

Acknowledgements/Funding statement

Financial support for the research, authorship, and publication of this article was received through a grant from the Bill & Melinda Gates Foundation [INV-010128]. The findings and conclusions contained within are those of the authors and do not necessarily reflect positions or policies of the Bill & Melinda Gates Foundation.

Patient involvement

No patients were involved

Conflicts of Interest

Having read and understood BMJ policy on declaration of interests, the authors have no conflict of interest to declare.

Licence

The Corresponding Author has the right to grant on behalf of all authors and does grant on behalf of all authors, an exclusive licence (or non-exclusive for government employees) on a worldwide basis to the BMJ Publishing Group Ltd ("BMJ"), and its Licensees to permit this article (if accepted) to be published in The BMJ's editions and any other BMJ products and to exploit all subsidiary rights, as set out in The BMJ's licence.

For peer review only

Abstract:

Introduction: The potential of timely, quality postnatal care (PNC) to reduce maternal and newborn mortality and to advance progress toward Universal Health Coverage (UHC) is well-documented. Yet, in many low- and middle-income countries, coverage of PNC remains low. Risk-stratified approaches can maximize limited resources by targeting mother-baby dyads meeting the evidence-based risk criteria which predict poor postnatal outcomes.

Objectives: To review evidence-based risk criteria for identification of at-risk mother-baby dyads, drawn from a literature review, and to identify key considerations for their use in a risk-stratified PNC approach.

Design/setting/participants: A virtual, semi-structured group discussion was conducted with maternal and newborn health experts on Zoom™. Participants were identified through purposive sampling based on content and context expertise.

Results: Seventeen experts, (5 male, 12 female), drawn from policymakers, implementing agencies and academia participated and surfaced several key themes. The identified risk factors are well-known, necessitating accelerated efforts to address underlying drivers of risk. Risk-stratified PNC approaches complement broader UHC efforts by providing an equity lens to identify the most vulnerable mother-baby dyads. However, these should be layered on efforts to strengthen PNC service provision for all mothers and newborns. Risk factors should comprise context-relevant, operationalizable, clinical and non-clinical factors. Even with rising coverage of facility delivery, targeted postnatal home visits still complement facility-based PNC.

Conclusion: Risk-stratified PNC efforts must be considered within broader health systems strengthening efforts. Implementation research at the country level is needed understand feasibility and practicality of clinical and non-clinical risk factors and identify unintended consequences.

For peer review only

Strengths and limitations of this study

- A major strength of this study is the depth and breadth of expertise of the participants in PNC, each bringing a combination of clinical, research, policy, and implementation skills across multiple low-and-middle-income countries.
- The consultation brought together experts, many of whom had engaged in parallel discussions around the topic, with the aim of advancing consensus on the role of a targeted PNC approach, and the key considerations of such an approach.
- However, the consultation included a limited number of global experts and did not include mothers, service providers, or experts representing Ministries of Health or other government stakeholders as ultimate custodians of a targeted PNC approach.
- In addition, nearly all experts came from a clinical background, which shaped perspectives shared.
- The discussion platform did not allow for confidentiality, which could have led to social desirability bias.

For peer review only - http://bmjopen.bmj.com/site/about/guidelines.xhtml

On the road to universal coverage of postnatal care: Considerations for a targeted postnatal care approach for at-risk mother-baby dyads in low- and middle-income countries informed by a consultation with global experts

***Angela Muriuki and colleagues** argue that there is a critical role for targeted postnatal care (PNC) approaches that prioritise mother-baby dyads who are at risk of poorer outcomes in the postnatal period, given the current low coverage of PNC. However, these approaches must be nested within existing strategies to strengthen provision of PNC for all mothers and babies rather than as stand-alone interventions.*

Introduction

Approximately 66% of maternal deaths and 75% of neonatal deaths occur within the first week after delivery.[1] WHO recommends postnatal care (PNC) at a facility, within 24 hours after birth, regardless of place of birth, observation within a facility for at least 24 hours after delivery, and early postnatal home visits (PNHVs) by community health workers (CHWs) to complement facility-based PNC.[2] Despite an increase in facility delivery, PNC coverage in many low- and middle-income countries (LMICs) remains below 50%.[3] In many LMICs, observation within a facility for 24 hours after delivery is challenging. This is in part due to pressure from families to leave after an uncomplicated delivery, lack of staffing and infrastructure for inpatient care, facility opening and closing times and a significant proportion of home deliveries.[4,5]

Evidence from LMICs with high newborn mortality rates demonstrates that early, quality PNHVs, within 72 hours after birth, can reduce newborn deaths by between 30-61% through support for healthy postnatal practices and early identification of danger signs and referral.[6] Yet high coverage of PNHVs is difficult to achieve in most LMICs, particularly due to limited coverage of CHW cadres.[7]

However, where adequate human resources are made available, evidence demonstrates benefit in identifying and providing risk-stratified PNHVs to mother-baby dyads.[8] Such an approach would identify and prioritize at-risk mother-baby dyads at the facility and at home for early PNHVs using evidence-informed criteria to identify those at risk of an adverse outcome.[9,10] Criteria can be clinical (e.g., medical conditions and complications) or nonclinical (e.g. sociodemographic, household, environmental factors). Using these criteria, health providers categorize mother-baby dyads based on risk and proactively create client-specific care plans.[11] A limited number of nascent program experiences have provided initial results and

lessons,[12] buttressed by a review of PNHV approaches that identified the need for "specifically targeting high-risk mothers and newborns for PNHVs, rather than using a 'blanket approach' that attempts to reach all mothers and newborns".[13] Yet the overall field lacks consensus around the need for a risk-stratified PNC approach, and the essential considerations for such an approach. Further, evidence from other fields of medicine has shown that a narrow focus using a risk-stratified approach could lead to unintended negative consequences including missing clients with no identifiable risk factors and potential for stigmatization.[14,15]

To inform the development and implementation of a risk-stratified PNC approach in LMICs, an iterative scoping literature review to identify risk criteria and an expert consultation were conducted. This paper presents the findings and recommendations from the expert consultation; findings from the scoping review will be published separately.

Methodology

A team of maternal and newborn health (MNH) experts, selected for their PNC expertise and drawn from academia, implementation partners and donors, were invited for a facilitated virtual expert consultation in April 2021. The consultation aimed to:

1. Review key risk factors, drawn from the literature review, for use at service delivery point (facility, community) to identify at-risk mother-baby dyads.
2. Identify key considerations to prioritize risk factors and operationalize a risk-stratified PNC approach.

A discussion guide was developed in line with the two key objectives, pretested with an MNH expert who was not part of the consultation and used to facilitate the meeting. Discussion questions were high-level to encourage engagement:

1. In your experience, what are the major risk factors, both proximate and distal, that predict poor outcomes in the postnatal period for both mother and baby?
2. What key issues or considerations should be taken into account when selecting risk factors for use in a risk stratification approach in different contexts?

The consultation was held on Zoom™ for ninety minutes. Consent was sought from the participants to record the proceedings and use the recordings while ensuring that all participant information was de-identified. An inductive analysis process was used, and data were coded into emerging themes following transcription.

Findings

For peer review only

Sixteen MNH experts participated in the consultation. The discussion mainly explored key considerations for prioritization and operationalization. The findings are presented along the key themes that emerged during the discussion.

Risk factors identified from the literature review

The risk factors identified from the iterative scoping literature review[1] (Textbox 1) were presented for the experts to reflect on and identify any additional factors based on their research and experience.

> **Textbox 1: Factors associated with poor outcomes for mothers and newborns in the postnatal period *(full list is presented in the scoping review paper)***
>
> Proximate factors include maternal age (<20, >35), primiparity and grand multiparity, shorter birth intervals, first order/rank neonates, male neonates, birth weight (smaller and larger than average), multiple gestation, previous history of death of child <5 years, and lack of or inadequate antenatal care
>
> Distant factors include low levels of parental education (lower than primary), parental employment (no employment or informal employment), rural residence, low household income, use of solid fuels and lack of clean water

The risk factors presented have been known to the MNH community for decades. The participants raised the importance of strengthening initiatives that address and eliminate these risk factors in addition to applying them for screening purposes. Additionally, they identified the role of broader, emerging issues such as climate change, conflict, displacement, and disease outbreaks in aggravating the proximate and distant risk factors which puts a larger proportion of mother-baby dyads at risk.

Key considerations for the operationalization of a risk-stratified PNC approach

a) Framing risk-stratified PNC approaches in the context of universal health coverage

Achieving universal health coverage (UHC) for PNC means providing quality, timely, accessible, equitable services for all mother-baby dyads, regardless of place of birth. Thus, it is critical to understand how a PNC approach that prioritises a sub-set of mothers and babies contributes to these aims. The journey towards achieving UHC is incremental and equity-focused, creating opportunities for risk-stratified PNC approaches that identify and prioritise those already facing poorer outcomes.

---

[1] The scoping review focused on population-based studies and excluded hospital-based studies and therefore the criteria identified were mainly non-clinical rather than the clinical risk factors traditionally used to screen for risk.

For peer review only - http://bmjopen.bmj.com/site/about/guidelines.xhtml

A risk-stratified PNC approach still requires a strengthened health system that can provide optimal PNC services, as the selected quotes in Textbox 2 illustrate. This includes strengthened provider capacity in PNC; adequate supply of essential medicines and equipment; strong referral systems including community follow-up; timely, reliable, quality data for risk screening; functional monitoring systems to assess functionality of the risk-stratified PNC approach and the provision of respectful, dignified care.

Textbox 2: Selected quotes from participants on framing risk stratified approaches within the context of UHC

‘we've been wondering whether focused approach and risk-stratified approach for the babies at most risk would be a more efficient way of doing it because our universal approach as you know, has been very challenging. It would be important to discuss this risk stratified approach but at the same time, you know balancing the universal approach, I think, somehow being able to do both will be important,’ ***Participant 3, F***

‘If you are looking at this risk factor I go back to the skills. Do they know how to identify this woman who is at risk, do they know how to deal with a woman who is at risk?’ ***Participant 15, F***

‘There are so many things that's tied to it [risk screening] like data to screen and to track morbidity and outcomes….and then the women's experience of care, and often that's forgotten….’ ***Participant 12, F***

b) Framing risk-stratified early PNHVs in the context of rising coverage of facility delivery

A benefit of the risk-stratified PNC approach is to prioritise limited community-level resources towards early PNHVs for at-risk mother-baby dyads. The rising coverage of facility deliveries and the missed opportunities to provide quality early PNC at facility level raised questions on whether a community-based risk-stratified PNC approach is still relevant and if more emphasis should be placed on quality facility-level PNC.

Despite the rising global coverage of facility delivery, a significant proportion of mothers still deliver at home in many LMICs, and many are discharged before the recommended 24 hours. Again, some categories of at-risk mother-baby dyads such as adolescent mothers or mothers with small and sick newborns will still require PNHVs even with strengthened, quality PNC services at facility level. Textbox 3 provides select expert quotes that illustrate this point.

For peer review only

Textbox 3: Selected quotes from participants on framing early postnatal home visits in the context of rising coverage of facility delivery.

'I think, personally, facility delivery is increasing and there are a lot of issues at facility level. I think, ideally, we should focus on improving the quality of services provided to mother and baby at facility level… increasingly I think what we really need is a strategy that addresses quality at the facility,' ***Participant 1, M***

''I think we are seeing more and more women deliver in the facility, but we are not seeing a reduction in [postnatal] mortality due to quality issues. If we could improve the quality of care during childbirth and have those who are at risk stay longer, we may see a return on investment in saving mothers' and newborns' lives,' ***Participant 14, F***

c) Selection of type of risk factors to use in a screening approach

There is value in including non-clinical risk factors in a screening approach. However, the challenges of their operationalization may be the reason why risk screening approaches have largely used clinical factors. For example, several of the factors identified are difficult to use for rapid screening at service delivery point by a health provider and could create stigma or embarrassment (e.g., household income). Some clinical risks can also be challenging to use in rapid screening (e.g., body mass index).

A tiered approach that begins with clinical risk factors, which are more acceptable and easier to use, and then includes the non-clinical risks could mitigate this challenge. Alternatively, selecting both clinical and non-clinical risks factors based on ease of use at service delivery level could address the challenge. Textbox 4 provides select quotes that illustrate this point.

Textbox 4: Selected quotes from participants on selection of risk factors for use.

'And yes, I do agree that, in addition to the clinical aspects of the risk factor, also looking at the other determinants like socio-economic elements that put a baby at risk, I think, are important also to include. Again, balancing all of this, you know so that it's programmable—that is the biggest challenge,' ***Participant 3, F.***

'May I suggest start with a clinical approach defined by context…,' ***Participant 13, M.***

'I like that idea of a tiered approach because starting with all the factors including the socioeconomic ones can be very difficult, so the suggestion of a tiered approach would work well.' ***Participant 6, F.***

d) Mitigating negative unintended consequences

Every pregnancy is a high-risk event. Many mothers and babies who develop complications in the postnatal period lack identifiable risk factors, and a risk-stratified approach should also rapidly identify and manage them. Risk-stratified PNC approaches must be nested within PNC strengthening initiatives so that the broader system acts as the safety net that catches those without identifiable risk factors and, thus, do not meet the screening criteria.

As one expert noted, improvements in overall quality and use of PNC by all women, including those not identified as at-risk, have been seen in areas where risk-stratified PNC approaches were used, highlighting the potential of a knock-on effect with implications for strengthening PNC for all women. As illustrated by the selected quotes in Textbox 5, this points towards a potential inherent risk mitigation factor that should be studied further.

Textbox 5: Selected quotes from participants on mitigating negative unintended consequences

'Certainly risk stratification is crucial and being able to identify moms and babies, who are more likely to have poor outcomes. I think we also know that sometimes those poor outcomes come from nowhere for both the mother and the baby. I feel like we need to consider also what a dual strategy is so that there's a specific strategy that deals with the mothers and babies who are more at risk and more likely to have those poor outcomes. And then, a broader based community strategy that can detect those issues that seem to come from nowhere for mothers and babies who don't appear to have any risk factors, but then subsequently develop significant issues,' ***Participant 10, F***

'What was found in one study is by initially concentrating on that risk stratification that indeed it led to improvements in PNC numbers, quality and content overall so you know again that kind of speaks to the theory of by concentrating on one aspect all boats rise…' ***Participant 6, F***

Discussion

Timely and quality postnatal care is critical for mothers and newborns. Yet in LMICs, PNC coverage remains stubbornly low[16] despite increased facility delivery. Prior risk stratification efforts have sought to identify and prioritize at-risk mothers during pregnancy.[17,18] Yet limited efforts have targeted at-risk mother-baby dyads during the postnatal period,[19] and little global consensus around the need for a risk-stratified PNC approach, and the considerations for such an approach, exists. Given the risk of stigma resulting from labelling mothers as "at-risk", the term "targeted PNC" may be more suitable for real-world application than "risk stratification" and is thus used throughout this discussion.

For peer review only - http://bmjopen.bmj.com/site/about/guidelines.xhtml

The expert consultation concluded that concurrent efforts are needed to target coverage of PNC to those most at risk of adverse outcomes, while improving quality of PNC to meet the increasing coverage of facility delivery. Through providing an equity lens to guide systematic identification of those most vulnerable to poor postnatal outcomes, targeted PNC should be considered a contribution—not an alternative—to UHC efforts. PNC approaches targeting those most at-risk of mortality in the postnatal period also contributes to the attainment of the 3rd Sustainable Development Goal.

We suggest that targeted PNC can be advanced in parallel, and as a contribution, to UHC efforts. In the short term, community-based provider cadres must be sufficiently resourced and staffed to allow screening of all mother-baby dyads, adequate counselling on danger signs, timely identification and outreach to at-risk mother-baby dyads, and rapid identification and referral for those who later develop complications. In the medium term, universal coverage of PNHVs can only be achieved when CHW-to-household ratios are fully adequate, and transportation is available for CHWs to reach assigned households; this requires advocacy with government to deepen investments in CHWs. Targeted PNHVs would be phased out as an adequate CHW-to-household ratio is reached and blanket PNHV coverage can be achieved. Longer-term investments are needed to address gaps in physical infrastructure and human resources, as well as social challenges that limit use of facility-based services, degrade service quality, and discourage longer stays. Further, while ANC coverage is generally higher,[20] efforts to strengthen coverage and quality of ANC are needed in tandem to improve detection of at-risk mother-baby dyads and encourage continuity of care.

Targeted PNC should be considered and provided in the presence of certain conditions. First, targeted PNC is only appropriate in the context of efforts to strengthen the timing and quality of facility PNC, including pre-discharge PNC, for all mother-baby dyads. This allows for identification and timely service provision for those who develop complications even in the absence of identifiable risk factors. Second, monitoring systems must allow both timely identification of mother-baby dyads meeting established risk criteria, and proactive tracking, identification and resolution of any unintended consequences.

Implementing a targeted PNC approach nested within broader equity-based UHC efforts entails consideration of how limited resources can be most effectively and efficiently targeted to those most likely to benefit. Exploration of several key considerations through robust country learning agendas is needed. First, decisions of which mother-baby dyads should be targeted should be guided by identification of risk factors comprising both clinical and non-clinical

For peer review only

predictors of poor outcomes. Evidence-based risk criteria for both facility- and community-based providers must be determined with consideration of both contextual relevance and feasibility of operationalization.

Next are considerations of how to operationalize selected evidence-informed clinical and non-clinical risk factors by facility and community providers. The timing of risk identification merits further consideration (i.e., some factors may be identifiable during pregnancy, while others manifest only following delivery). Clear and feasible guidance on actions to be taken for mother-baby dyads meeting risk criteria is needed and must be developed with careful consideration of the implications for provider workload and motivation, client flow, and facility infrastructure capacity. Given the vulnerability of at-risk mother-baby dyads, particularly those with identified non-clinical risks, efforts to increase accessibility and ensure respectful care are particularly critical elements of broader UHC efforts. Unintended consequences—positive and negative impacts on the health system and on health outcomes—must be assessed, monitored continuously, and addressed in consultation with health workers and policymakers. Further, efforts are needed to gather perspectives of mothers, their families, and communities to understand the acceptability of a targeted PNC approach and to identify unintended consequences from clients' perspectives.

Notably, broader efforts are needed to reduce prevalence of underlying clinical and non-clinical risk factors that contribute to poor maternal and newborn outcomes. Mitigating the non-clinical risk factors will require a multi-sectoral effort beyond the health system.

This consultation has several limitations. The expert consultation invited perspectives of a small number of global and country experts. While the format facilitated robust engagement of experts with deep and diverse expertise in the subject matter, and involvement in strategy and policy from the organizational to global levels, findings represent the perspectives of a small and targeted sample. While care was taken to ensure diversity of experts' sex, organisation affiliation, and country of origin, perspectives of other relevant stakeholders are not represented. Notably, all experts came from a clinical background, which shaped perspectives shared. The discussion explored high-level policy considerations, and did not explore acceptability of targeted PNC from the perspectives of mothers, families, or health workers. The discussion did not allow for confidentiality, which could have led to social desirability bias.

Conclusion

For peer review only

Targeted community-based PNC approaches, nested within broader efforts to strengthen quality PNC services including pre-discharge PNC, could improve outcomes for mother-baby dyads most at-risk of morbidity and mortality during the postnatal period.

References

[1] World Health Organization. Postnatal care of the mother and newborn [Internet]. Geneva; 2013. Available from: http://apps.who.int/iris/bitstream/10665/97603/1/9789241506649_eng.pdf.

[2] World Health Organization. Postnatal care of the mother and newborn [Internet]. Geneva; 2013. Available from: http://apps.who.int/iris/bitstream/10665/97603/1/9789241506649_eng.pdf.

[3] Victora, CG, Requejo, JH, Barros, AJD, Berman, P, Bhutta, Z, Boerma T. Countdown to 2015: a decade of tracking progress for maternal, newborn, and child survival. Lancet, The. 2016;387(10032):1049–59.

[4] Bashar Mda, Aggarwal A, Pilania M. Factors causing early maternity discharge of uncomplicated normal delivery cases at government health facilities in Northern India: An exploratory study. J Educ Health Promot [Internet]. 2020;9(1):198. Available from: http://www.jehp.net/text.asp?2020/9/1/198/293940

[5] Campbell OMR, Cegolon L, Macleod D, Benova L. Length of Stay After Childbirth in 92 Countries and Associated Factors in 30 Low- and Middle-Income Countries: Compilation of Reported Data and a Cross-sectional Analysis from Nationally Representative Surveys. Myers JE, editor. PLOS Med [Internet]. 2016 Mar 8;13(3):e1001972. Available from: https://dx.plos.org/10.1371/journal.pmed.1001972

[6] Gogia S, Sachdev HS. Home visits by community health workers to prevent neonatal deaths in developing countries: a systematic review. Bull World Health Organ [Internet]. 2010 Sep 1;88(9):658-666B. Available from: http://www.who.int/bulletin/volumes/88/9/09-069369.pdf

[7] Lassi ZS, Das JK, Salam, RA et al. Evidence from community level inputs to improve quality of care for maternal and newborn health: interventions and findings. Reprod Health. 2014. 11, S2. https://doi.org/10.1186/1742-4755-11-S2-S2

[8] McPherson R, Hodgins S. Postnatal home visitation: Lessons from country programs operating at scale. J Glob Health [Internet]. 2018 Jun;8(1). Available from: http://jogh.org/documents/issue201801/jogh-08-010422.pdf

[9] World Health Organization (WHO). Risk approach for maternal and child health care. In: World Health Forum. 1981. p. 413–22.

[10] Ochejele S, Ijiko E, Obulu M. Assessment of the risk approach in the reduction of maternal mortality in north-central Nigeria. J West African Coll Surg [Internet]. 2011;1(2):76–85. Available from: http://www.ncbi.nlm.nih.gov/pubmed/25452955%0Ahttp://www.pubmedcentral.nih.gov/articlerender.fcgi?artid=PMC4170261

[11] Mora J, Iturralde MD, Prieto L, Domingo C, Gagnon M-P, Martínez-Carazo C, et al. Key aspects related to implementation of risk stratification in health care systems-the ASSEHS study. BMC Health Serv Res [Internet]. 2017 Dec 5;17(1):331. Available from: http://bmchealthservres.biomedcentral.com/articles/10.1186/s12913-017-2275-3

[12] Maternal and Child Survival Program. Risk Stratification for Prioritized Postnatal Care in Rural India: An innovative approach for improving quality of care for mothers and newborns. 2020. Unpublished technical brief.

[13] Hodgins S, McPherson R, Kerber K. Postnatal Care, with a Focus on Home Visitation: A Design Decision-Aid for Policymakers and Program Managers. Maternal and Child Survival Program. Available from: https://www.healthynewbornnetwork.org/hnn-content/uploads/PostnatalCarewithaFocusonHomeVisitation.pdf

[14] Garg A, Boynton-Jarrett R, Dworkin PH. Avoiding the unintended consequences of screening for social determinants of health. JAMA - J Am Med Assoc. 2016;316(8):813–4.

For peer review only

[15] Feudtner C, Schall T, Nathanson P, Berry J. Ethical Framework for Risk Stratification and Mitigation Programs for Children With Medical Complexity. Pediatrics [Internet]. 2018 Mar 1;141(Supplement 3):S250–8. Available from: http://pediatrics.aappublications.org/lookup/doi/10.1542/peds.2017-1284J

[16] Victora, CG, Requejo, JH, Barros, AJD, Berman, P, Bhutta, Z, Boerma T. Countdown to 2015: a decade of tracking progress for maternal, newborn, and child survival. Lancet, The. 2016;387(10032):1049–59.

[17] Patel A, Prakash AA, Pusdekar Y V., Kulkarni H, Hibberd P. Detection and risk stratification of women at high risk of preterm birth in rural communities near Nagpur, India. BMC Pregnancy Childbirth. 2017;17(1).

[18] Ochejele S, Ijiko E, Obulu M. Assessment of the risk approach in the reduction of maternal mortality in north-central Nigeria. J West African Coll Surg [Internet]. 2011;1(2):76–85. Available from: http://www.ncbi.nlm.nih.gov/pubmed/25452955%0Ahttp://www.pubmedcentral.nih.gov/articlerender.fcgi?artid=PMC4170261

[19] World Health Organization. Postnatal care of the mother and newborn [Internet]. Geneva; 2013. Available from: http://apps.who.int/iris/bitstream/10665/97603/1/9789241506649_eng.pdf.

[20] Benova L, Tunçalp Ö, Moran AC, Campbell OMR. Not just a number: examining coverage and content of antenatal care in low-income and middle-income countries. *BMJ Global Health* 2018;**3:**e000779.

For peer review only

Standards for Reporting Qualitative Research (SRQR)*

http://www.equator-network.org/reporting-guidelines/srqr/

Title and abstract

	Page/line no(s).
**Title** - Concise description of the nature and topic of the study Identifying the study as qualitative or indicating the approach (e.g., ethnography, grounded theory) or data collection methods (e.g., interview, focus group) is recommended	Page 1/Line 4<br>Page 6/Line 96
**Abstract** - Summary of key elements of the study using the abstract format of the intended publication; typically includes background, purpose, methods, results, and conclusions	Page 4/Line 70

Introduction

**Problem formulation** - Description and significance of the problem/phenomenon studied; review of relevant theory and empirical work; problem statement	Page 7/Line 132
**Purpose or research question** - Purpose of the study and specific objectives or questions	Page 7/Line 137

Methods

**Qualitative approach and research paradigm** - Qualitative approach (e.g., ethnography, grounded theory, case study, phenomenology, narrative research) and guiding theory if appropriate; identifying the research paradigm (e.g., postpositivist, constructivist/ interpretivist) is also recommended; rationale**	Page 7/Line 142
**Researcher characteristics and reflexivity** - Researchers' characteristics that may influence the research, including personal attributes, qualifications/experience, relationship with participants, assumptions, and/or presuppositions; potential or actual interaction between researchers' characteristics and the research questions, approach, methods, results, and/or transferability	Page 7/Line 142<br>Page 7/Line 149
**Context** - Setting/site and salient contextual factors; rationale**	Page 6/Line 108
**Sampling strategy** - How and why research participants, documents, or events were selected; criteria for deciding when no further sampling was necessary (e.g., sampling saturation); rationale**	Page 7/Line 142
**Ethical issues pertaining to human subjects** - Documentation of approval by an appropriate ethics review board and participant consent, or explanation for lack thereof; other confidentiality and data security issues	Page 7/Line 156
**Data collection methods** - Types of data collected; details of data collection procedures including (as appropriate) start and stop dates of data collection and analysis, iterative process, triangulation of sources/methods, and modification of procedures in response to evolving study findings; rationale**	Page 7/Line 142

For peer review only

**Data collection instruments and technologies** - Description of instruments (e.g., interview guides, questionnaires) and devices (e.g., audio recorders) used for data collection; if/how the instrument(s) changed over the course of the study	Page 7/Line 149
**Units of study** - Number and relevant characteristics of participants, documents, or events included in the study; level of participation (could be reported in results)	Page 8/Line 162
**Data processing** - Methods for processing data prior to and during analysis, including transcription, data entry, data management and security, verification of data integrity, data coding, and anonymization/de-identification of excerpts	Page 7/Line 158
**Data analysis** - Process by which inferences, themes, etc., were identified and developed, including the researchers involved in data analysis; usually references a specific paradigm or approach; rationale**	Page 7/Line 158
**Techniques to enhance trustworthiness** - Techniques to enhance trustworthiness and credibility of data analysis (e.g., member checking, audit trail, triangulation); rationale**	n/a

Results/findings

**Synthesis and interpretation** - Main findings (e.g., interpretations, inferences, and themes); might include development of a theory or model, or integration with prior research or theory	Page 8/Line 176
**Links to empirical data** - Evidence (e.g., quotes, field notes, text excerpts, photographs) to substantiate analytic findings	Page 9/Textbox 2<br>Page 10/Textboxes 3-4<br>Page 11/Textbox 5

Discussion

**Integration with prior work, implications, transferability, and contribution(s) to the field -** Short summary of main findings; explanation of how findings and conclusions connect to, support, elaborate on, or challenge conclusions of earlier scholarship; discussion of scope of application/generalizability; identification of unique contribution(s) to scholarship in a discipline or field	Page 11/Line 228
**Limitations** - Trustworthiness and limitations of findings	Page 13/Line 296

Other

**Conflicts of interest** - Potential sources of influence or perceived influence on study conduct and conclusions; how these were managed	Page 3/Line 58
**Funding** - Sources of funding and other support; role of funders in data collection, interpretation, and reporting	Page 3/Line 49

For peer review only - http://bmjopen.bmj.com/site/about/guidelines.xhtml

*The authors created the SRQR by searching the literature to identify guidelines, reporting standards, and critical appraisal criteria for qualitative research; reviewing the reference lists of retrieved sources; and contacting experts to gain feedback. The SRQR aims to improve the transparency of all aspects of qualitative research by providing clear standards for reporting qualitative research.

**The rationale should briefly discuss the justification for choosing that theory, approach, method, or technique rather than other options available, the assumptions and limitations implicit in those choices, and how those choices influence study conclusions and transferability. As appropriate, the rationale for several items might be discussed together.

Reference:

O'Brien BC, Harris IB, Beckman TJ, Reed DA, Cook DA. **Standards for reporting qualitative research: a synthesis of recommendations.** *Academic Medicine*, Vol. 89, No. 9 / Sept 2014 DOI: 10.1097/ACM.0000000000000388

For peer review only

For peer review only - http://bmjopen.bmj.com/site/about/guidelines.xhtml
