# Supplementary material for: On the road to universal coverage of postnatal care: considerations for a targeted postnatal care approach for at-risk mother–baby dyads in low-income and middle-income countries informed by a consultation with global experts
Source: BMJ Open. 2022 Jun 14;12(6):e058408. doi: 10.1136/bmjopen-2021-058408 (PMC9198691; doi:10.1136/bmjopen-2021-058408)
Supplement: Reviewer comments [file bmjopen-2021-058408.reviewer_comments.pdf]

PEER REVIEW HISTORY

BMJ Open publishes all reviews undertaken for accepted manuscripts. Reviewers are asked to complete a checklist review form (**http://bmjopen.bmj.com/site/about/resources/checklist.pdf**) and are provided with free text boxes to elaborate on their assessment. These free text comments are reproduced below.

ARTICLE DETAILS

**TITLE (PROVISIONAL)**	On the road to universal coverage of postnatal care: Considerations for a targeted postnatal care approach for at-risk mother-baby dyads in low- and middle-income countries informed by a consultation with global experts
**AUTHORS**	Muriuki, Angela; Yahner, Melanie; Kiragu, Michael; de Graft-Johnson, Joseph; IZULLA, PRESTON

VERSION 1 – REVIEW

**REVIEWER**	Schaaf, Marta<br>Independent Consultant
**REVIEW RETURNED**	22-Nov-2021

**GENERAL COMMENTS**	This paper is straightforward, well written, and pretty comprehensive given the limitations of the study design. The issue addressed is important, and the focus on the feasibility of implementation is important. However, I do not feel comfortable with the idea that a 90 minute Zoom consultation is sufficent data to produce a peer-reviewed manuscript. I suggest that the authors might consider: mutliple consultations with regional focus; re-orienting the paper so that it includes the full report of the scoping review and the consultation; and/or make an evidence-based argument for the approach described, such as by reviewing how often these questions of feasibility are systematically assessed and included in priority setting and decision-making. (That being said, I do no think the Zoom consultation itself can be described as systematic).

**REVIEWER**	Shibanuma, Akira<br>The University of Tokyo Graduate School of Medicine Faculty of Medicine, Department of Community and Global Health
**REVIEW RETURNED**	24-Nov-2021

**GENERAL COMMENTS**	This study was conducted to identify the relevance, feasibility, and operational issues in implementing targeted postnatal care in low- and middle-income countries through expert interviews. The manuscript is highly informative by summarizing experts' views on the current issues in the provision of postnatal care and possible issues and consequences of implementing targeted postnatal care. This manuscript should be widely disseminated among policymakers, experts, service providers as well as clients and community leaders who are actively working for maternal, newborn, and child health issues. I hope the following points would improve the quality of the manuscript.

	1. The experts who joined the discussion seem to be working for the designers and suppliers' side of maternal, newborn, and child health, not from the demand side. Since this study identified non-clinical issues (or social determinants) of postpartum health and postnatal care provisions, viewpoints from the demand side, such as pregnant women, mothers, and community leaders may also be important. If this understanding is correct, the authors may want to clarify that the scope of the discussion is basically from the designers and suppliers' side, and that acceptability of targeted postnatal care from the demand side was not examined.<br>2. The authors mentioned in the Introduction section that "Where high coverage of postnatal home visits is difficult to achieve due to these challenges, as in most LMICs, evidence demonstrates benefit in identifying and providing postnatal home visits to mother – baby dyads who face a higher risk of poor outcomes." (Lines 67-) In contrast, the part of the conclusion is that "Targeted PNC approaches should be considered in tandem with and layered on complementary efforts aiming to strengthen the coverage, timing, and quality of facility PNC for all mother-baby dyads rather than as stand-alone interventions." (Lines 337-) The authors seem not to discuss how targeted postnatal approach can be achieved in parallel with improving the coverage and quality of universal-based postnatal care under the resource-limited setting. The authors may want to add a paragraph in the Discussion section for this issue.<br>3. Related to the comment above, is targeted postnatal care, particularly home-visit postnatal care, resource-saving, in comparison with the provision of postnatal care at the facility level? At a primary level health facility, the number of nurses and community health workers tends to be small. Some facilities have only one or two health workers. It would make service provisions at the facility difficult while health workers conduct home-visit postnatal care. Home-visit postnatal care requires the means of transportation, such as motorbikes, which also need fuel costs. The authors may want to address the trade-off between facility-based service provision and home-visit postnatal care under the resource-limited setting.<br>4. It is unclear if the saturation was achieved after the discussion among 17 experts. The authors may need to justify why the number of experts involved in the discussion was sufficient.<br>5. For better detection of risks among women and newborn to identify at-risks for targeted postnatal care, the coverage and quality of antenatal care and delivery assistance are important under the continuum of care. While inadequate antenatal care was identified in the scoping review (Textbox 1), it was not discussed in the Discussion section. It would be nice to consider addressing the importance of antenatal care for better detection of at-risk women and newborns.<br>6. While experts address shorter stay at a health facility after childbirth, the importance of longer stay after childbirth was not addressed in the Discussion section. To shift resources for targeted postnatal care, the resources for universal postnatal care should be saved. Longer stay after childbirth might be an important option to improve the coverage of postnatal care while health facility infrastructure, security, and nightshift for health workers are essential to make longer stay possible.

VERSION 1 – AUTHOR RESPONSE

Reviewer 1
1. This paper is straightforward, well written, and pretty comprehensive given the limitations of the study design. The issue addressed is important, and the focus on the feasibility of implementation is important. However, I do not feel comfortable with the idea that a 90 minute Zoom consultation is sufficent data to produce a peer-reviewed manuscript. I suggest that the authors might consider: mutliple consultations with regional focus; re-orienting the paper so that it includes the full report of the scoping review and the consultation; and/or make an evidence-based argument for the approach described, such as by reviewing how often these questions of feasibility are systematically assessed and included in priority setting and decision-making. (That being said, I do no think the Zoom consultation itself can be described as systematic).
2. We greatly appreciate the concerns raised around the study design. We have revised the manuscript to more clearly explain that the consultation effectively served as a culmination of parallel discussions, bringing together experts to collaboratively discuss a nascent topic in the field. While additional consultations are infeasible at this time, we feel that there is value in sharing the perspectives and recommendations emerging from the consultation. We have also expanded both the Introduction (beginning on Line 129) and the Limitations sections (beginning on Line 296) to further clarify the purpose and the limitations of this consultation.

Reviewer 2
1. The experts who joined the discussion seem to be working for the designers and suppliers' side of maternal, newborn, and child health, not from the demand side. Since this study identified non-clinical issues (or social determinants) of postpartum health and postnatal care provisions, viewpoints from the demand side, such as pregnant women, mothers, and community leaders may also be important. If this understanding is correct, the authors may want to clarify that the scope of the discussion is basically from the designers and suppliers' side, and that acceptability of targeted postnatal care from the demand side was not examined.
We have noted the need to gather perspectives of mothers, community members, and service providers as an important future area for learning in the Discussion (beginning on Line 288), and have noted this in the Limitations section (beginning on Line 304).

2. The authors mentioned in the Introduction section that "Where high coverage of postnatal home visits is difficult to achieve due to these challenges, as in most LMICs, evidence demonstrates benefit in identifying and providing postnatal home visits to mother – baby dyads who face a higher risk of poor outcomes." (Lines 67-) In contrast, the part of the conclusion is that "Targeted PNC approaches should be considered in tandem with and layered on complementary efforts aiming to strengthen the coverage, timing, and quality of facility PNC for all mother-baby dyads rather than as stand-alone interventions." (Lines 337-) The authors seem not to discuss how targeted postnatal approach can be achieved in parallel with improving the coverage and quality of universal-based postnatal care under the resource-limited setting. The authors may want to add a paragraph in the Discussion section for this issue.
We agree, and have expanded and reorganized the Discussion section (beginning on Line 245) to discuss how targeted PNC can be advanced in parallel to UHC efforts.

3. Related to the comment above, is targeted postnatal care, particularly home-visit postnatal care, resource-saving, in comparison with the provision of postnatal care at the facility level? At a primary level health facility, the number of nurses and community health workers tends to be small. Some facilities have only one or two health workers. It would make service provisions at the facility difficult while health workers conduct home-visit postnatal care. Home-visit postnatal care requires the means of transportation, such as motorbikes, which also need fuel costs. The authors may want to address

the trade-off between facility-based service provision and home-visit postnatal care under the resource-limited setting.
As the reviewer notes, current gaps in physical and human resources as well social challenges that that make it difficult to have longer stays require more resources and are long-term investment. In the short term, we see greater investments in CHWs as necessary to bridge the gap and provide a minimum standard of coverage, which also advances progress toward UHC as reflected in the comment above. We have revised the Discussion section to reflect this (beginning on Line 254).

4. It is unclear if the saturation was achieved after the discussion among 17 experts. The authors may need to justify why the number of experts involved in the discussion was sufficient.
The number reflects the constraints related to a virtual discussion, but was overall adequate due to experts' experience in the subject matter, their involvement at policy and strategy level within their organizations and at global level, as well as their diverse expertise within the subject area i.e., research, clinical practice. Further, the consultation essentially served as a culmination of parallel and ongoing discussions on the topic. We have revised the Introduction (beginning on Line 129) and Limitations (beginning on Line 296) sections to better articulate the aims and scope of the consultation, and to clarify that our saturation refers only to the information provided by the 17 experts.

5. For better detection of risks among women and newborn to identify at-risks for targeted postnatal care, the coverage and quality of antenatal care and delivery assistance are important under the continuum of care. While inadequate antenatal care was identified in the scoping review (Textbox 1), it was not discussed in the Discussion section. It would be nice to consider addressing the importance of antenatal care for better detection of at-risk women and newborns.
We agree that antenatal care is an important entry point for identifying mothers who may face complications, and have included discussion of the importance of addressing gaps in coverage and quality of antenatal care in the Discussion section (beginning on Line 256).

6. While experts address shorter stay at a health facility after childbirth, the importance of longer stay after childbirth was not addressed in the Discussion section. To shift resources for targeted postnatal care, the resources for universal postnatal care should be saved. Longer stay after childbirth might be an important option to improve the coverage of postnatal care while health facility infrastructure, security, and nightshift for health workers are essential to make longer stay possible.
We agree that addressing underlying factors that limit duration of facility stay following delivery is an important consideration, and have added discussion of the considerations that limit duration of stay following delivery in the Discussion section (beginning on Line 252)

VERSION 2 – REVIEW

**REVIEWER**	Schaaf, Marta<br>Independent Consultant
**REVIEW RETURNED**	21-Mar-2022

**GENERAL COMMENTS**	The authors have responded to the questions posed by the two reviewers. The article reads more clearly and better acknowledges the limitations of the study design. However, I stand by my initial concern that publishing this separately from the scoping review is unfortunate. Unless the scoping review findings are vast, it would better help the field to put these consultation findings in dialogue with the scoping review findings, rather than publishing them as two separate manuscripts.

**REVIEWER**	Shibanuma, Akira<br>The University of Tokyo Graduate School of Medicine Faculty of Medicine, Department of Community and Global Health
**REVIEW RETURNED**	30-Mar-2022

**GENERAL COMMENTS**	Thank you very much for addressing all the comments from the reviewer made in the previous round of the peer-review process. The strength and limitations of this study were made clear as a result of the revision.
